# Development of GALDI-FT-ICR-MS Methods for the Analysis of Xylan Oligomers

**DOI:** 10.1021/acs.biomac.5c01386

**Published:** 2025-10-28

**Authors:** Klara Sander, Jan Zuber, Erica Brendler, Carla Vogt

**Affiliations:** 26545 Institute of Analytical Chemistry, TU Bergakademie Freiberg, Lessingstrasse 45, Freiberg 09599, Germany

## Abstract

Xylans, a highly abundant type of hemicelluloses in plant-based biomass, are currently being investigated as a renewable substitute for fossil resources. Thus, understanding their properties and structure is crucial for efficient utilization. This study aims to develop analytical methods for characterizing the soluble and insoluble compounds of xylans, using graphite-assisted laser desorption/ionization combined with Fourier-transform ion cyclotron resonance mass spectrometry (GALDI-FT-ICR-MS). A beechwood xylan was utilized as a representative sample for this work. Infrared (IR) and nuclear magnetic resonance (NMR) spectroscopy were additionally used to obtain further structural information. The results from the MS analyses indicate that liquid- and solid-state GALDI-MS techniques can aid in the characterization of the xylan molecules, and both ionization modes were found to be essential for a comprehensive understanding of xylans. The findings of this study may contribute to a deeper understanding of the structural characteristics of xylans and assist in the efficient application of these polysaccharides.

## Graphical abstract

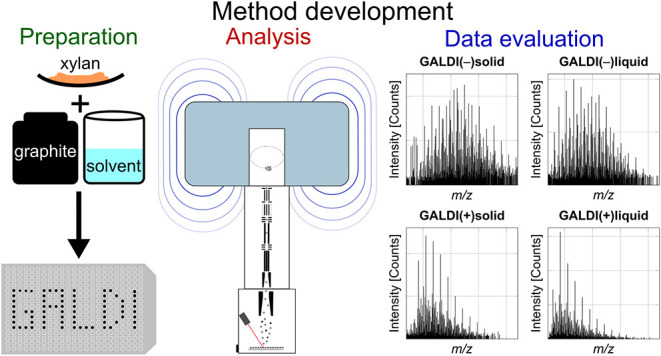

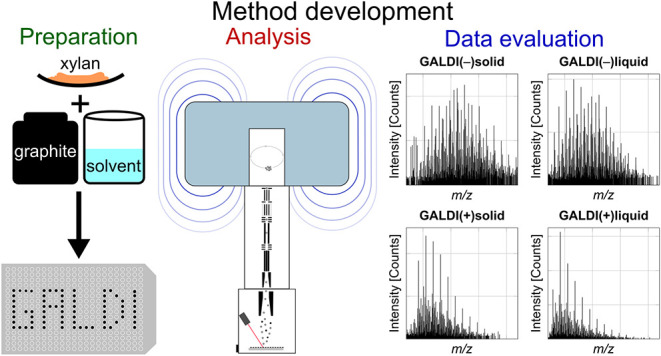

## Introduction

Plant cell walls mainly consist of polysaccharides and proteins, and in lignified plants also lignins. Polysaccharides represent the primary component of cell walls, with hemicelluloses comprising the second most abundant type of polysaccharides therein, following celluloses.^1,2^ Hemicelluloses are linear heteropolymers composed of pentose and hexose sugars, including xylose, arabinose, glucose, and mannose. The classification of hemicelluloses is dependent on their primary sugar unit, resulting in a differentiation into xyloglucans, xylans, mannans, glucomannans, and glucans.^1,3^ Xylans thereby represent a significant hemicellulose type in plant cell walls and, consequently, in plant-based biomass. They make up to 20 to 30% of the secondary cell walls in dicots, including hardwoods such as beechwood, whereas in monocots, including grasses, xylans make up to 20% of the primary cell walls.^2,3^ The backbone of xylan polymers in terrestrial plants is composed of β-D-xylose monomers linked by β-1,4-glycosidic bonds. The main chain can additionally be branched with other sugar units or derivatives and, in some cases, could also be partly acetylated. The substitution pattern of the xylose backbone is strongly dependent on the origin of the xylan, resulting in a differentiation in homoxylans, arabinoxylans, glucuronoxylans, and arabinoglucuronoxylans or glucuronoarabinoxylans.^2,4^ Xylans originating from hardwood, such as the one used in this study, mainly contain substitutions with 4-O-methylglucuronic acid most likely at position C_2_ of the xylose monomer, labeling them as glucuronoxylans. Their main chain commonly also shows partial acetylation.^5^

In recent years, biopolymers such as xylans gained a lot of attention due to being regarded as environmentally friendly, regenerative, biodegradable, and nontoxic alternatives to fossil resources.^1^ Xylans possess a high application potential, for example, in the chemical industry as raw materials for the production of basic chemicals such as furfural, ethanol, or lactic acid.^4^ Another potential field of application is the generation of polymeric films that can be utilized as biodegradable packaging or membrane materials.^1,2,4^ In the pharmaceutical industry, hemicelluloses also show great potential for applications, such as their use as excipients in pharmaceutical formulations as an alternative to cellulose-based materials, because hemicelluloses are, unlike celluloses, digestible for humans.^6^ Additionally, a considerable number of fields of research exist, including biofuels, nanoparticles, and hydrogels, which are thoroughly described and discussed in numerous articles.^2,4,7–9^ A notable challenge in this regard is the high complexity of lignocellulosic biomass and the variability of hemicellulose composition and structure, which are dependent on their origin and the isolation process.^3,10^ In order to utilize these compounds to their fullest potential and identify new applications for these abundant and inexpensive substances, it is therefore necessary to understand their properties and, consequently, their structure.

The structural characterization of polysaccharides, including hemicelluloses, presents certain challenges due to the complexity of their structure, which originates from varying monosaccharide compositions, linkage patterns, branching, and other factors.^11^ Therefore, various analytical techniques are often employed for the characterization of these biopolymers. In this regard, for example, chromatographic methods such as size exclusion chromatography, liquid chromatography, or gas chromatography are frequently utilized for the determination of the molar mass or a preliminary separation of the oligo- or monosaccharides to support the structural characterization of polysaccharides.^10–12^ Furthermore, nuclear magnetic resonance spectroscopy (NMR) and high-resolution mass spectrometry (HRMS) are important techniques for the structural elucidation of biopolymers.^11^ Thereby polysaccharides present challenging sample systems for the analysis using mass spectrometry due to their usually very low ionization efficiency resulting from a low number of easily ionizable functional groups.^6,13^ The primary technique used for the analysis of polysaccharides in the field of HRMS is a combination of matrix-assisted laser desorption/ionization with time-of-flight mass analyzers (MALDI-TOF-MS).^6,10,11,13–17^ It is often employed for the determination of the molar mass, the molar mass distribution, and the analysis of linkage types and substitution patterns in polysaccharides.^6,10,13,14,18,19^ Additionally, atmospheric pressure ion sources, such as electrospray ionization (ESI), are often applied for biopolymer analyses.^5,12,20^ In addition to TOF-MS, ion trap mass spectrometers such as Fourier-transform ion cyclotron resonance mass spectrometer (FT-ICR-MS) or linear trap quadrupol (LTQ) devices can also be used to determine the structural composition of biopolymers.^17,18,21^

One crucial point in MALDI-MS is the choice of matrix. For the characterization of polysaccharides, 2,5-dihydroxybenzoic acid (2,5-DHB) is one of the most commonly used matrices.^17,22^ Other well-established matrices that are utilized include α-cyano-4-hydroxycinnamic acid (CHCA) and 2,4,6-trihydroxyacetophenone (THAP). However, research is ongoing for new matrices, such as binary matrices, nanoparticles, and carbon-based matrices.^22^ In previous studies, our research group demonstrated the applicability of high-purity graphite as an alternative to commonly used matrices in a variety of sample systems. It was already successfully used for the analysis of pyrolysis oils from different sources, such as scrap tires,^23^ coals,^24–26^ and pyrolysis wax and oil from plastics recycling^27^ or other complex mixtures.^28^ In addition, graphite-assisted laser desorption/ionization (GALDI) has been employed in the analysis of biological samples, encompassing the characterization of extracellular polymeric substances (EPS) of *Didymosphenia geminata* ^29^ and in the analysis of lignins.^30,31^ This demonstrates the versatility of GALDI, indicating its capacity to characterize a wide array of solid and liquid sample systems. It is, therefore, promising that GALDI-MS can also aid in the characterization of hemicelluloses.

The goal of this study was the development of preparation and analysis routines for the characterization of soluble and insoluble components of hemicelluloses with GALDI-FT-ICR-MS. Xylans were selected as the representative system for hemicelluloses due to their abundance in plant-based biomass.^32^ The method development was conducted on a commercial beechwood xylan and was based on methods previously developed by our research group for the analysis of different lignin samples.^30^ By adjustment of important parameters for the sample preparation and the mass spectrometric parameters, these methods were successfully applied to the characterization of the beechwood xylan sample. The analyses revealed structurally different homologous series of xylan compounds, indicating a substitution of the xylose main chain with 4-O-methylglucuronic acid and a partial acetylation, which is typical for hardwood xylans.^4^ To obtain further structural information, nuclear magnetic resonance (NMR) and infrared (IR) spectroscopy were utilized, supporting the mass spectrometric findings. It is therefore hoped that the developed methods and the generated results will contribute to a deeper understanding of the structural peculiarities of hemicelluloses and thus will help to establish them as alternatives to fossil resources.

## Experimental Section

### Chemicals

For the development of the analysis methods, a commercial xylan originating from beechwood from Carl Roth GmbH was used. The high purity graphite powder was purchased from Micro to Nano (purity 99.9%, particle size 5 μm). Triethylamine (NEt_3_, Carl Roth GmbH, purity ≥ 99.5%), chloroform (CHCl_3_, Merck KGaA-LiChrosolv, purity ≥ 99.8%), *n*-hexane (Carl Roth GmbH, purity ≥ 98%), toluene (Carl Roth GmbH, purity ≥ 99.8%), ammonium acetate (NH_4_Ac, VWR Chemicals, purity 99.1%), tetrahydrofuran (THF, VWR Chemicals, purity ≥ 99.7%), isopropanol (i-PrOH, VWR Chemicals, purity 100%), sodium trifluoroacetate (NaTFA, Sigma-Aldrich, purity ≥ 99%), ammonium trifluoroacetate (NH_4_TFA, Sigma-Aldrich, purity ≥ 99%), methanol (MeOH, Supelco, purity 99.9%), trifluoroacetic acid (TFA, Merck Chemicals, purity 99.8%), ammonia (NH_3_, Applichem, concentration 32%), sodium hydroxide (NaOH, Applichem, concentration 32%), glycerol (Alfa Aesar, purity 99%), sodium acetate (NaAc, Grüssing GmbH, purity 99%), sodium carbonate (Na_2_CO_3_, Fluka Chemicals, purity >99.0%), calcium hydroxide (Ca­(OH)_2_, Carl Roth GmbH, purity ≥ 96%) and dimethyl sulfoxide (DMSO, TH. Geyer GmbH, purity ≥ 99.8%) were utilized as purchased.

### Sample Preparation

A more detailed description of the development of the preparation and analysis routines for the solid- and liquid-state GALDI­(+/-)-FT-ICR-MS methods can be found in the “Results and Discussion” section and in Section S1. Thus, only the optimized routines will be described for the positive and negative ionization modes in the following.

For the liquid-state analyses, the sample was extracted using sonication in an ultrasonic bath with DMSO as the solvent. The extraction duration and temperature were set to 15 min and 30 °C. The xylan concentration for extraction was 10 g L^–1^. For MS analyses in negative ionization mode, an analyte suspension, consisting of 100 μL of extract, 100 μL of a 100 mM NH_3_ solution in THF, 20 mg of high-purity graphite powder, and 15 μL of glycerol, was prepared. For the positive ionization mode, 100 μL of extract was suspended in 100 μL of a 10 mM NaTFA solution in methanol and 30 mg graphite.

The solid-state analyses in negative ionization mode were conducted using a suspension of a 1:10 (w/w) mixture of the solid sample and high-purity graphite, with a total amount of solids of 30 mg in 200 μL of a 5 mM NH_3_ solution in a 50:50 (v/v) mixture of THF and DMSO, and 15 μL of glycerol. For the positive ionization mode, a 1:10 (w/w) mixture of the solid sample and high-purity graphite, with a total amount of solids of 50 mg, was suspended in 200 μL of a 50 mM NH_4_TFA solution in a 20:80 (v/v) mixture of CHCl_3_ and DMSO.

The suspensions were sonicated for 10 min at room temperature. Then, 1.0 μL (GALDI­(+/-) liquid and GALDI(−)­solid) or 0.8 μL (GALDI­(+)­solid) of the suspension were spotted on a stainless steel MALDI target (MTP 384 ground steel) and dried in a drying oven for 20 min at 40 °C.

### GALDI-FT-ICR-MS

All mass spectrometric experiments were conducted on a 15 T solariX FT-ICR-MS from Bruker Daltonics, which is equipped with both an ESI and a MALDI source. The MALDI source contains a Smart Beam II laser, which is a frequency-tripled Nd:YAG laser with a wavelength of 355 nm, a pulse duration of 3 ns, a pulse energy of 500 μJ, a peak power of 170 kW, and an average power of 1.5 W. For all analyses, the resolution was *R* = 800,000 at an *m*/*z* of 400. The resulting data sets had a size of 8 M. The mass spectrometric data were all collected in triplicate. The most important parameters for the ion source and FT-ICR-MS, which were used according to the results of the method development process, are summarized in the following table (Table 1).

**Table 1:** Summary of the Mass Spectrometric Parameters for the GALDI-MS Analyses after Method Development.

parameters	positive ion mode	negative ion mode
scan range	153.49 to 5,000.00 Da	
plate offset voltage	40.0 V	–60.0 V
deflector plate voltage	200.0 V	–180.0 V
number of laser shots	20	
laser power	25% (liquid)	35% (liquid)
	33% (solid)	34% (solid)
laser frequency	500 Hz	
laser focus	ultralarge	
number of scans	32 or 512	
time-of-flight (TOF)	0.9 ms (liquid)	1.3 ms (liquid)
	1.2 ms (solid)	1.4 ms (solid)
transfer exit lens	10.0 V	–10.0 V
analyzer entrance	5.0 V	–5.0 V
side kick	–0.0 V	–0.0 V
side kick offset	3.0 V	–3.0 V
front trap plate	–0.800 V	0.800 V
back trap plate	–0.800 V	0.800 V
sweep excitation power	19.0%	
free induction decay (FID)	2.7962 s	

### Data Processing

The data processing was in general carried out according to Sander et al.^30^ A short summary will be given hereinafter due to sample-related changes in the data handling process. Calibration of the GALDI-FT-ICR-MS data sets was performed using in-house-generated calibration lists for xylans. For the primary data handling, Bruker Daltonics software DataAnalysis 5.0 (SR1) was used to generate peak and molecular formula lists. Molecular formula assignment was carried out using peaks with a signal-to-noise ratio (S/N) ≥ 10, and the variance from the theoretical mass should not exceed 0.3 ppm. The elemental composition was defined within the boundaries of C_c_H_h_O_o_Na_na_ with c: 0 ≤ *c* ≤ 100, h: 0 ≤ *h* ≤ 200, and o: 0 ≤ *o* ≤ 100 for both ionization modes, and na: 0 ≤ na ≤ 3 for the positive ionization mode (na = 0 for the negative ionization mode). Further data processing was performed using MATLAB 2023b from Mathworks utilizing in-house scripts for blank correction, molecular formula filtering (applying the rules from Herzsprung et al.,^33,34^ ) data evaluation, and plotting.

### FT-IR Spectroscopy

The IR analyses were conducted on a Nicolet iS10 FT-IR spectrometer using attenuated total reflection (ATR) equipped with a diamond prism (30,000 to 200 cm^–1^). Therefore, the solid powdered sample was applied directly to the ATR unit and pressed on with the pressure tower. It was analyzed using a wavenumber range of 4,000 to 400 cm^–1^, a number of scans of 32 and a resolution of 4 cm^–1^. The acquisition and processing of the IR spectra were performed using the software OMNIC (version 9.8.372) by Thermo Fisher Scientific.

### NMR Spectroscopy

^13^C solid-state NMR analyses were performed on a 400 MHz BRUKER AVANCE III HD WB spectrometer equipped with a 4 mm cross-polarization/magic angle spinning (CP/MAS) probe. The analyses were conducted at 100.67 MHz (^13^C) and 400.3 MHz (^1^H) as well as a spinning speed of 12.5 kHz using 4 mm ZrO_2_ rotors. For CP, a contact time of 1 ms and a 50% ramp on the ^1^H channel were applied. 20,480 scans were accumulated with a recycling delay of 3 s as well as an acquisition time of 35 ms. The acquisition, processing, and the following analysis of the spectra was performed using Topspin (version 4.0.6 and 3.6.5) by Bruker Biospin.

## Results and Discussion

### Method Development−Liquid-State GALDI-FT-ICR-MS

Mass spectrometry is a widely used method for the structural characterization of polysaccharides and hence also for hemicelluloses. It is thereby often accompanied by a rather time-consuming sample pretreatment, such as extraction, degradation, or derivatization (e.g., methylation).^11,12^ In order to comprehensively characterize the structural composition of untreated hemicelluloses using mass spectrometry, it is necessary to develop new analysis and preparation routines, which was the goal of this study. The group of xylans was selected as a representative for hemicelluloses because they are one of the most abundant types of hemicelluloses in plant-based biomass.^2^ The starting point of our work was analysis routines for GALDI(−)-FT-ICR-MS developed previously by our research group for the analysis of lignins.^30^ During method development, the criteria used to select the most suitable options for sample preparation and mass spectrometric parameters generally included, besides the spectral appearance, the values for peak number, total ion current (TIC), or mean *m*/*z*. The peak number describes the number of signals that exhibit an S/N ratio of at least 5. All of these parameters were determined from the blank-corrected data sets. It should be noted that this section focuses on the essential steps of the method development process. For a comprehensive overview of all method development steps, readers are referred to Section S1.

In this study, the initial focus was on the development of routines for the analysis of the soluble compounds present in the deployed beechwood xylan. Therefore, the xylan sample was extracted using an ultrasonic bath. To obtain reliable results, this extraction was optimized in terms of extraction solvent (optimum: DMSO), concentration (optimum: 10 g L^–1^), duration (optimum: 15 min), and temperature (optimum: 30 °C). In a second step, the GALDI sample preparation was optimized for the positive and negative ionization modes. The sample preparation includes the suspension of high-purity graphite in the xylan extract and an additional solvent (cosolvent, negative: THF, positive: MeOH).

It was known from our previous work^24–26,30^ that the amount of graphite used to form the sample suspension plays an important role for the ionization process, which is why this parameter was optimized for this sample system (optimum negative: 20 mg, positive: 30 mg). Additionally, the influence of ionization supplements was investigated to support the formation of ions in the polysaccharide sample. It was shown that the addition of bases for the negative ionization mode (optimum: NH_3_) and organic salts in the positive ionization mode (optimum: NaTFA) indeed supports the ionization of polysaccharide molecules. In this regard, the required supplement concentration (optimum negative: 50 mM, positive: 5 mM) was also optimized. The further method development process also revealed that for the negative ionization mode, the addition of glycerol has a positive effect on the spot quality, whereas for the positive ionization mode, glycerol should not be added due to miscibility issues.

The final step of method development was to optimize the mass spectrometric parameters, resulting in a time-of-flight (TOF) of 1.3 ms for the negative ionization mode and 0.9 ms for the positive ionization mode. The TOF thereby describes the time between the ejection of the ions from the collision cell and their capture in the ICR cell.

### Method Development−Solid-State GALDI-FT-ICR-MS

In order to further simplify the preparation routines, an investigation was conducted to ascertain whether the extraction could be omitted, which additionally enabled the characterization of insoluble xylan oligomers. Therefore, the powdered sample was directly mixed with high-purity graphite and then suspended in the solvent mixture. For the solid-state analyses, the sample-to-graphite ratio (S/G ratio) plays a major role for the ionization process, which is why this parameter had to be optimized during method development. It was found that an S/G ratio of 1:10 (w/w) is the most appropriate for further investigations for both ionization modes, with a total amount of solids of 30 mg for the negative and 50 mg for the positive ionization mode.

In order to achieve a good comparability between the solid- and liquid-state methods, an attempt was made to use the same solvent mixtures as those utilized for the liquid-state analyses. This was successful for the negative ionization mode (DMSO/THF (50:50 v/v)). For the positive ionization mode, a mixture of CHCl_3_/DMSO with a CHCl_3_/DMSO ratio of 20:80 (v/v) was deployed in order to ensure a good spot quality. To further enhance the spot quality, the addition of glycerol is recommended for the negative ionization mode, while this approach proved ineffective for the positive ionization mode, as already outlined for the liquid-state analyses.

The remaining steps in the method development process were analogous to the liquid-state analyses in terms of ionization supplements (optimum: negative: NH_3_; positive: NH_4_TFA) and their concentration (optimum: negative: 5 mM; positive: 50 mM) as well as the settings for the TOF (optimum negative: 1.4 ms, positive: 1.2 ms). A more detailed overview of the development process is presented in Section S1.

### Comparison of the Developed Analysis Routines

In order to make a comparison between the developed analysis methods for GALDI­(+/-)­solid- and GALDI­(+/-)­liquid-FT-ICR-MS and to obtain structural information about the analyzed beechwood xylan, analyses were conducted using 512 scans. The blank-corrected and averaged mass spectra for the four developed analysis routines are presented in Figure 1, while the corresponding values for the peak number and mean *m*/*z* are visualized in Figure 2.

**Figure 1:**
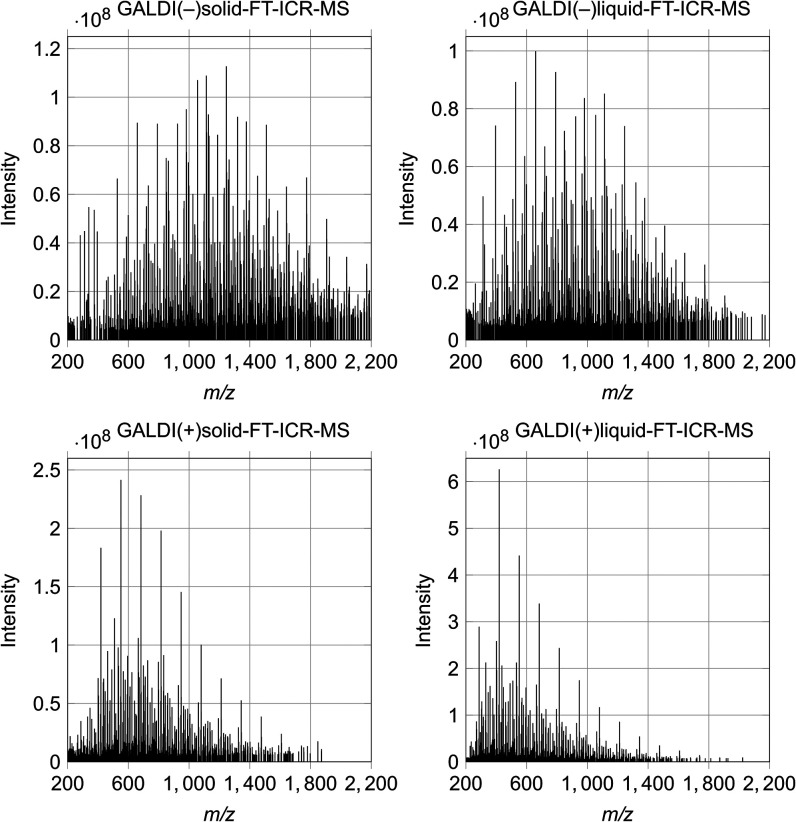
Mass spectra of the beechwood xylan sample analyzed with liquid- and solid-state GALDI-FT-ICR-MS in both positive and negative ionization modes.

**Figure 2:**
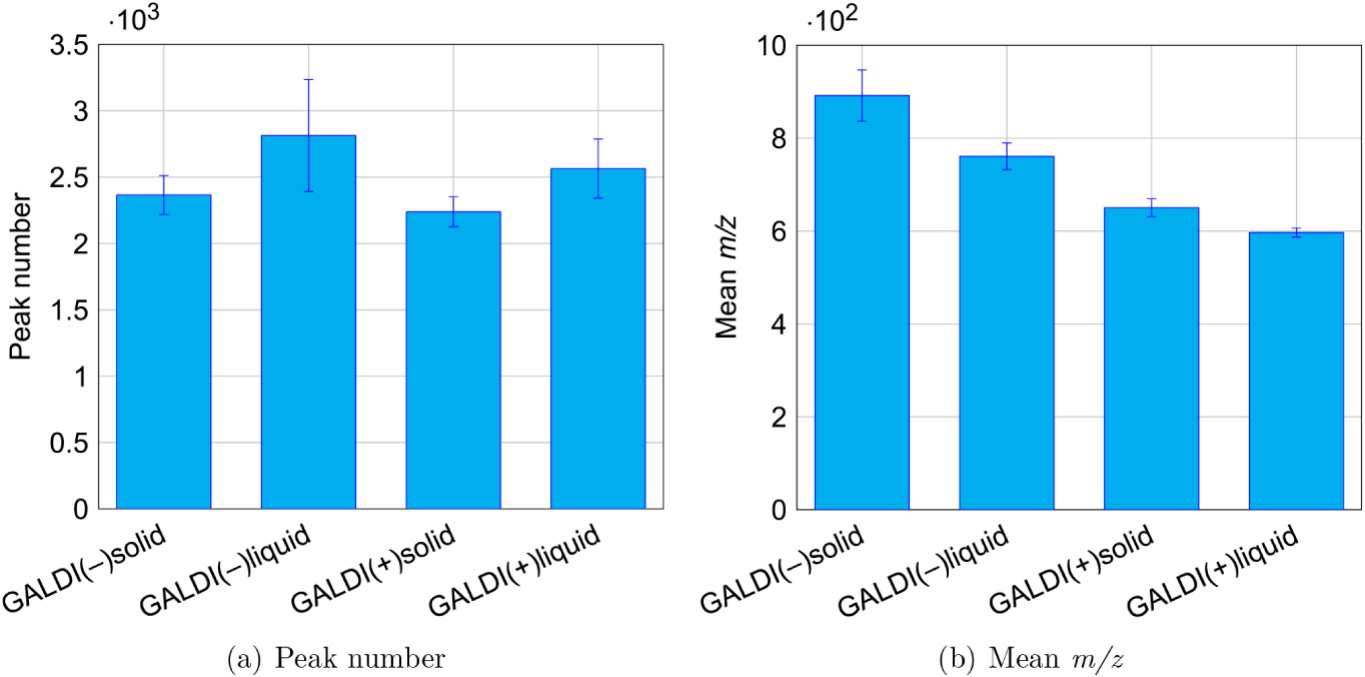
Comparison of the mass spectrometric results for the four optimized analysis routines: GALDI(−)­solid, GALDI(−)­liquid, GALDI­(+)­solid, and GALDI­(+)­liquid.

The mass spectra show that for the positive ionization mode, the *m*/*z* range covered by the sample signals is smaller (solid: 200 to 1,800, liquid: 200 to 1,700) than for the negative ionization mode (solid: 200 to 2,200, liquid: 200 to 2,000). This can mainly be explained by the differences in the TOF used, which is significantly smaller in positive ionization mode (see Table 1). That results in a center of mass at lower *m*/*z* values and a preference for signals at smaller *m*/*z* values compared with the negative ionization mode. A comparison of the covered *m*/*z* range for the solid- and liquid-state analyses reveals that the former is slightly larger than the latter. As would be anticipated from the mass spectra, the values for the mean *m*/*z* also exhibit a similar pattern (see Figure 2b), which means that a larger quantity of longer oligomers is present in the negative ionization mode and for the solid-state analysis methods. Moreover, the mass spectra show a signal pattern that is characteristic of samples containing oligomers of different sizes. Hereby, the signal pattern within one ionization mode is rather similar, although the spectral features display a slightly greater variability between the ionization modes. This signal pattern indicates the presence of various series of signals characterized by an *m*/*z* spacing of approximately 132, which is associated with xylose monomers (C_5_H_8_O_4_), suggesting the presence of xylooligomer series with differing structural compositions. As illustrated in Figure 2a, the peak number for all four methods falls within the standard deviation limits at comparable values. This indicates that a comparable number of different ion types can be formed during ionization.

A further notable observation in the mass spectra is the variation in signal intensity between the positive and negative ionization modes. Specifically, the positive ionization mode displays a considerably higher signal intensity compared to the negative ionization mode. This can be attributed to the tendency of polysaccharides, such as xylans, to form positively charged adducts, such as those with sodium ions ([M+Na]^+^), rather than forming negatively charged ions by deprotonation ([M–H]^−^). This results in a higher ionization efficiency in the positive ionization mode and thus leads to higher signal intensities.^35^

In order to obtain information regarding the reproducibility of the four developed methods, the relative standard deviation (RSD) for the peak number and the mean *m*/*z* was determined, as demonstrated in Table 2. Furthermore, the RSD values for the TIC are presented in Section S2. The RSD was calculated using eq 1, where σ is the standard deviation and x is the absolute mean value for the respective parameter.

1 $$\mathrm{RSD}=\frac{\sigma }{x}\times 100\%$$

In this case, two different approaches were utilized. First, the spot-to-spot homogeneity was investigated by performing a triple determination on a single sample, measuring on three spots along a row of spots on the MALDI target (described as ″1 preparation″ in Table 2). Second, the reproducibility of the preparation methods was examined by performing three individual sample preparations and a single determination for each prepared sample, with a single spot per row of spots on the MALDI target measured (described as ″3 preparations″ in Table 2).

**Table 2:** Summary of the Values for the Relative Standard Deviation (RSD) of the Mean *m/z* and the Peak Number for the Four Developed Methods Determined via Two Different Approaches.

method	parameter	1 preparation	3 preparations
GALDI(−)solid	mean *m*/*z*	4.58%	6.18%
	peak number	5.17%	6.21%
GALDI(−)liquid	mean *m*/*z*	6.17%	3.76%
	peak number	4.49%	14.98%
GALDI(+)solid	mean *m*/*z*	5.49%	2.93%
	peak number	6.48%	5.00%
GALDI(+)liquid	mean *m*/*z*	2.27%	1.60%
	peak number	4.83%	8.70%

As demonstrated in Table 2, the RSD values for a single preparation are consistently below 10% and are comparable for all four methods. This indicates satisfactory spot-to-spot homogeneity for the developed preparation and analysis routines. Furthermore, the results obtained for the three separate preparations are also promising. In this case, the values for the solid-state methods remain comparable for both mean *m*/*z* and peak number, while for the liquid-state methods, only the RSD values for the mean *m*/*z* remain similar. Conversely, the RSD for the peak number shows a notable increase, especially for negative ionization mode. This increase can be attributed to the influence of human factors, which have a greater impact on the variability of analyses obtained from three separate preparations as opposed to a single one. It is further relevant to acknowledge the complexity of the sample system under examination, which is characterized by polysaccharides exhibiting low ionization efficiencies. This leads to irregular ionization and therefore to variations among analyses. Overall, the RSD values for the three preparations indicate a reliable reproducibility of the preparation methods, with an RSD not exceeding 15% for mean *m*/*z* and peak number.^36^

The results show successful method development and indicate that the most significant differences exist between the ionization modes, whereas the solid-state and liquid-state analyses within one ionization mode appear to provide results that are relatively comparable.

### Statistical Evaluation of Molecular Formulas

To gain more detailed insight into the data obtained, the blank-corrected and averaged molecular formula lists were analyzed, in addition to the peak list evaluation. In the first step, the similarity between the different methods was investigated. Figure 3 shows a correlation matrix illustrating the correlation of the relative peak intensities among the four methods, with ρ being the Pearson correlation coefficient. The results demonstrate a high positive correlation between the solid-state and liquid-state analyses within one ionization mode (ρ_pos_ = 0.80 and ρ_neg_ = 0.82), whereas between the ionization modes, there is only a low correlation. With that, the assumptions made from peak list evaluation could be confirmed, indicating a high similarity between the molecular formulas resulting from solid- or liquid-state analyses. Conversely, the variation appears to be more pronounced between the ionization modes, as expected.

**Figure 3:**
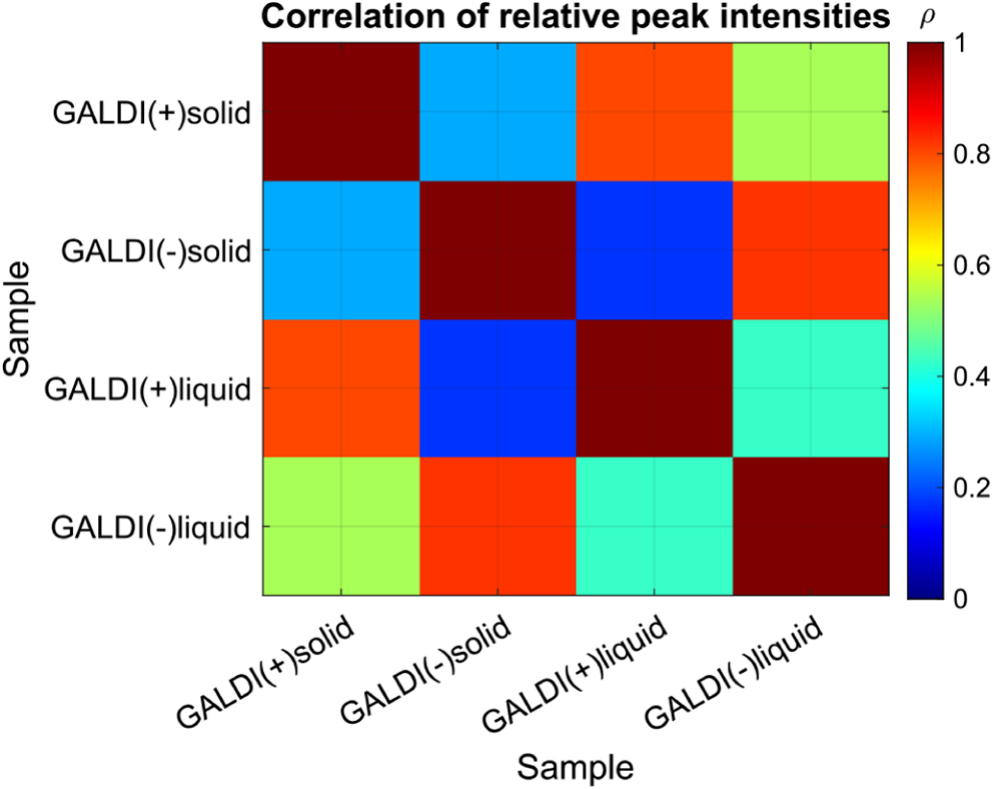
Correlation of the relative intensities of the molecular formulas for all four methods with a confidence interval of 99.9%. The correlation coefficient is represented by a color-coded scale, with blue showing no positive correlation and red indicating a high positive correlation.

To illustrate this further, Figure 4 shows the UpSet plot for the molecular formulas of the four methods.

**Figure 4:**
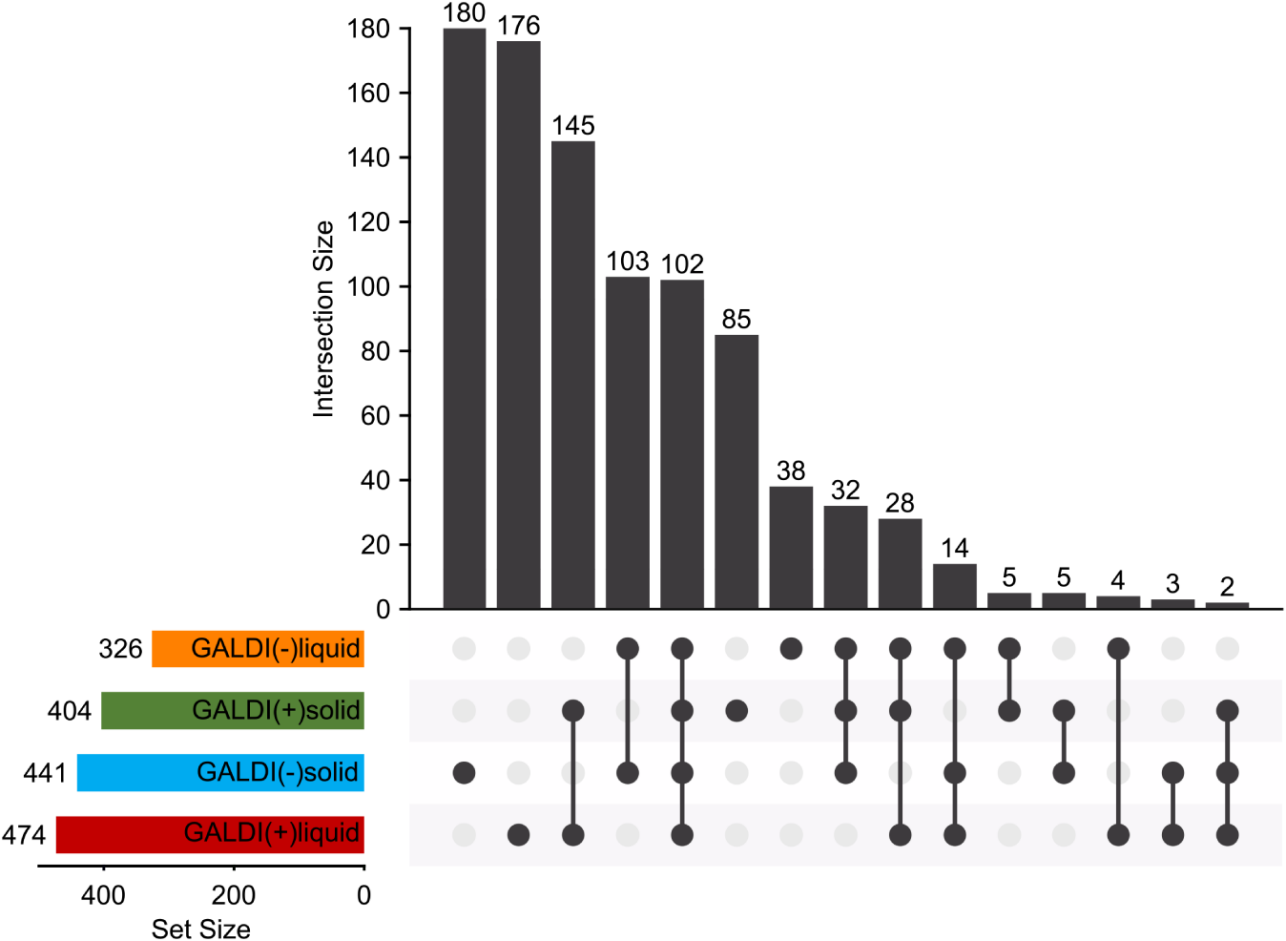
UpSet plot to facilitate a comparative analysis of all four analysis methods in order to evaluate the degree of similarity among the methods and to identify the common molecular formulas generated by each method.

Therein, it is evident that a relatively high number of molecular formulas are present exclusively for the GALDI(−)­solid (180) and GALDI­(+)­liquid (176) analysis methods, which indicates a certain diversity between the analyses. An examination of the corresponding *m*/*z* values reveals that for GALDI(−)­solid, primarily higher *m*/*z* values are exclusively found here, with 94.4% of the molecular formulas in this group exhibiting *m*/*z* values exceeding 1,000. Conversely, for GALDI­(+)­liquid, there are more smaller *m*/*z*, with 86.4% of them falling below 1,000. This is to be expected between the ionization modes due to the lower TOF values in positive ionization mode, which favor smaller *m*/*z* values, whereas in the negative ionization mode, higher *m*/*z* values are preferred. Furthermore, the discrepancy between the solid-state and liquid-state analyses within a single ionization mode can be attributed, to a certain extent, to variations in the TOF too. As demonstrated in Table 1, the TOF values for the solid-state analyses are marginally elevated in comparison to those for the liquid-state analyses. This discrepancy can potentially result in a shift in *m*/*z* preferences between methods, particularly in positive ionization mode. In the case of negative mode, it is also necessary to consider the greater difference in the total number of molecular formulas between GALDI(−)­solid and GALDI(−)­liquid. Nevertheless, it is visible that also a large quantity of molecular formulas is present in both solid-state and liquid-state analyses of one ionization mode (positive: 145 and negative: 103) and within all four methods (102). Overall, the intersection between the methods is therefore around one-third to one-half of the molecular formulas of the respective method. Therefore, these findings also suggest a notable degree of similarity between the methods employed for one ionization mode and a satisfactory degree of consistency across all four methods.

Another statistical approach is the clustering of the identified molecular formulas into different heteroatomic classes based on their oxygen content. For the beechwood xylan, a high number of different oxygen-containing classes with oxygen numbers of up to 70 (GALDI(−)­solid) could be detected, each containing a relatively small number of molecular formulas (see Figure S14 in Section S3). This pattern is indicative of the presence of diverse oxygen-containing oligomers that vary in size and structure. The addition of oxygen-containing monomers leads to an increase in the oxygen number, consequently resulting in a multitude of oxygen-containing classes. In accordance with the findings derived from the peak list results and mass spectra, the positive ionization mode methods are found to more likely yield a greater number of molecular formulas in the lower oxygen classes, while the negative ionization mode methods preferably lead to higher oxygen numbers. Within one ionization mode, the distribution of the oxygen-containing classes is similar (see Figure S14 in Section S3). A more detailed evaluation of the total number of molecular formulas per oxygen-containing class and the relative abundance of the heteroatomic classes (see Figure S15 in Section S3) is presented in Section S3.

The results suggest that both ionization modes are essential for providing a comprehensive characterization of xylans. Due to the high similarity between the solid- and liquid-state methods, it was decided to prioritize one of them. Given the reduced risk of altering the sample properties during preparation, the inability to exclude molecules due to their insolubility, the smaller preparation effort, and the slightly more favorable results, it was decided that the GALDI solid-state techniques should be preferably used for sample preparation.

### Structural Insights into the Xylan Oligomers

In addition to the general data evaluation and the numeric insights into the developed methods, the structural information obtained with these new methods is also significant. Due to the high similarity between the solid- and liquid-state methods, regarding the structural evaluation, only the data from the solid-state analyses in both ionization modes are presented below. The corresponding data for the liquid-state analyses are presented in Sections S4 and S7.

For a first classification of the molecular formulas obtained by GALDI­(+/-)-FT-ICR-MS the O/C and H/C ratios for every molecular formula can be calculated and visualized in van Krevelen plots, illustrated in Figures 5a and b.

**Figure 5:**
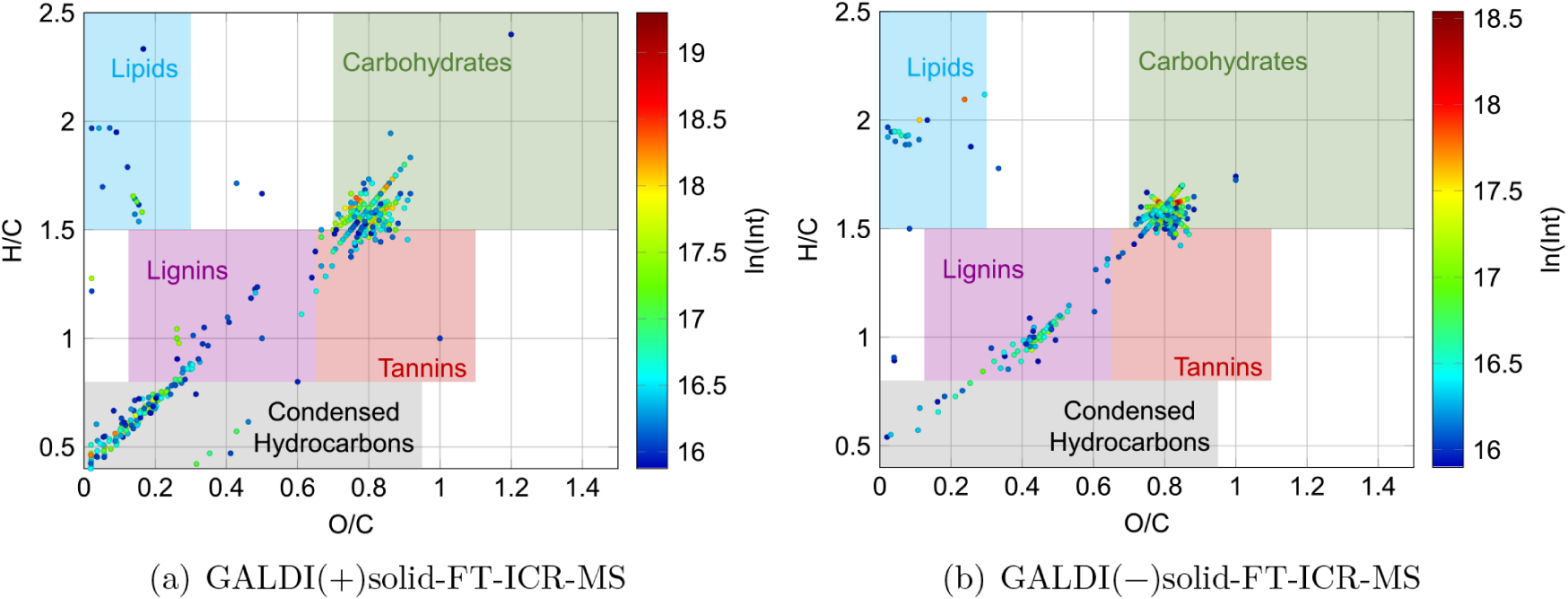
van Krevelen plots of the xylan sample analyzed using solid-state GALDI-FT-ICR-MS. The observed intensities are presented logarithmically and color-coded (blue: low intensity; green to yellow: medium intensity; red: high intensity). The boundaries for the compound class regions were defined according to Ayala-Ortiz et al^37^.

This approach can be utilized to estimate the potential compound classes to which the molecular formulas can be assigned, given the fact that specific O/C and H/C regions are characteristic of specific compound classes. The carbohydrate region is thereby typically located at high H/C ratios (1.5 to 2.5) and medium to high O/C ratios (0.7 to 1.5).^37^ As illustrated in Figure 5, for both ionization modes, a major part of the data points is located in the carbohydrate region. An overview of the percentage of the molecular formulas in the different compound classes can be found in Figure S17 and Table S2 in Section S4. It is evident that the molecules of beechwood xylan exhibit comparatively low H/C and O/C ratios for carbohydrates. In addition to the data points corresponding to carbohydrates, there are data points that are located on a line in the direction of more aromatic compounds. The majority of these data points for the negative ionization mode are located in the region typical of lignin-like structures (H/C: 0.8 to 1.5, O/C: 0.125 to 0.65).^37^ In contrast, for the positive ionization mode, more data points in the condensed hydrocarbon region (H/C: 0.2 to 0.8, O/C: 0.0 to 0.95)^37^ were detected. It is plausible that these data points are derived from impurities in the sample, in addition to those present in the region characteristic of lipid-like structures (H/C: 1.5 to 2.5, O/C: 0.0 to 0.3).^37^ These impurities could be originating from other plant constituents or might have been generated during the production process and were not removed during the purification stage. A comparison of the positive and negative ionization modes reveals that the carbohydrate-like data points extend over a slightly broader area in the positive ionization mode. This phenomenon can be attributed to the enhanced ionization efficiency of carbohydrates in the positive ionization mode.

For further insight into the structure of the molecules, n_c_-DBE plots are suitable. They illustrate the double bond equivalent (DBE) as a function of the number of carbon atoms (n_c_). For the analyzed sample, these plots contain two main groups of data points (see Figure S18 in Section S5). The first one is characterized by relatively low values for the DBE, which increase linearly by one with an increase in the number of carbon atoms by five (see Figure S18 in Section S5). Additionally the number of oxygen atoms also rises linearly with the number of carbon atoms (see Figure S18 in Section S5). This phenomenon is characteristic of the addition of one cyclic xylose monomer to the oligomer chain, assigning these data points to the xylooligomers in the xylan sample. The second group of data points is located at higher n_C_ and DBE values (see Figure S18 in Section S5), indicating a higher degree of unsaturation. Consequently, these data points can be attributed to the ones located in the regions characteristic of condensed hydrocarbons (positive ionization mode) and lignin-like compounds (negative ionization mode) of the van Krevelen plots (see Figure 5). As previously described, these compounds could be assigned to impurities present in the sample. A more detailed evaluation of the n_c_-DBE plots is presented in Section S5.

Another approach for acquiring more detailed information about the structural composition and variety in the beechwood xylan sample is the use of RKM-*m*/*z* plots.^38–40^ These are variations of the Kendrick mass defect plot, in which the remainders of the Kendrick masses (RKM) are plotted against the *m*/*z*. The remainders of Kendrick mass can be calculated using eq 2.^38^ Herein, KM signifies the exact Kendrick mass of the molecule in question, M_nominal_(C_5_H_8_O_4_) denotes the nominal molar mass of the repeating unit (nominal *m*/*z* = 132), and the floor function corresponds to rounding the values to the next smaller whole number.^38^ As the repeating unit, a xylose monomer with the molecular formula C_5_H_8_O_4_ was chosen.

2 $$\mathrm{RKM}=\frac{\mathrm{KM}}{M_{\mathrm{nominal}}\left(C_{5}H_{8}O_{4}\right)}-\mathrm{floor}\left(\frac{\mathrm{KM}}{M_{\mathrm{nominal}}\left(C_{5}H_{8}O_{4}\right)}\right)$$

The resulting plots for both ionization modes are illustrated in Figures 6a and b. The data points located on a horizontal line thereby correspond to structurally similar oligomers that differ in their chain length. As is evident, a considerable number of horizontal lines are present at varying RKM values. These, in turn, represent a homologous series of oligomers that differ structurally. The high number of homologous series thereby indicates a high structural variety in beechwood xylan. To illustrate this further, Figures 6c and d shows the mass spectra of a selection of these homologous oligomer series in both ionization modes (a larger version of the mass spectra is presented in Figure S19 in Section S6). The associated data points are highlighted in the corresponding RKM-*m*/*z* plots. A structural proposal was formulated for the selected series based on the molecular formulas and DBE values. Furthermore, the proposal was aligned with the findings from IR and NMR analyses of the sample (see section ″IR and NMR analyses″) and results that are already known in the literature^5^ on the structure of beechwood xylans. However, it is crucial to acknowledge that all structural proposals must be verified through tandem MS analyses, as they are merely reasonable assumptions at this stage. The *m*/*z* ranges and corresponding structural proposals for both ionization modes are presented in Table 3.

**Figure 6:**
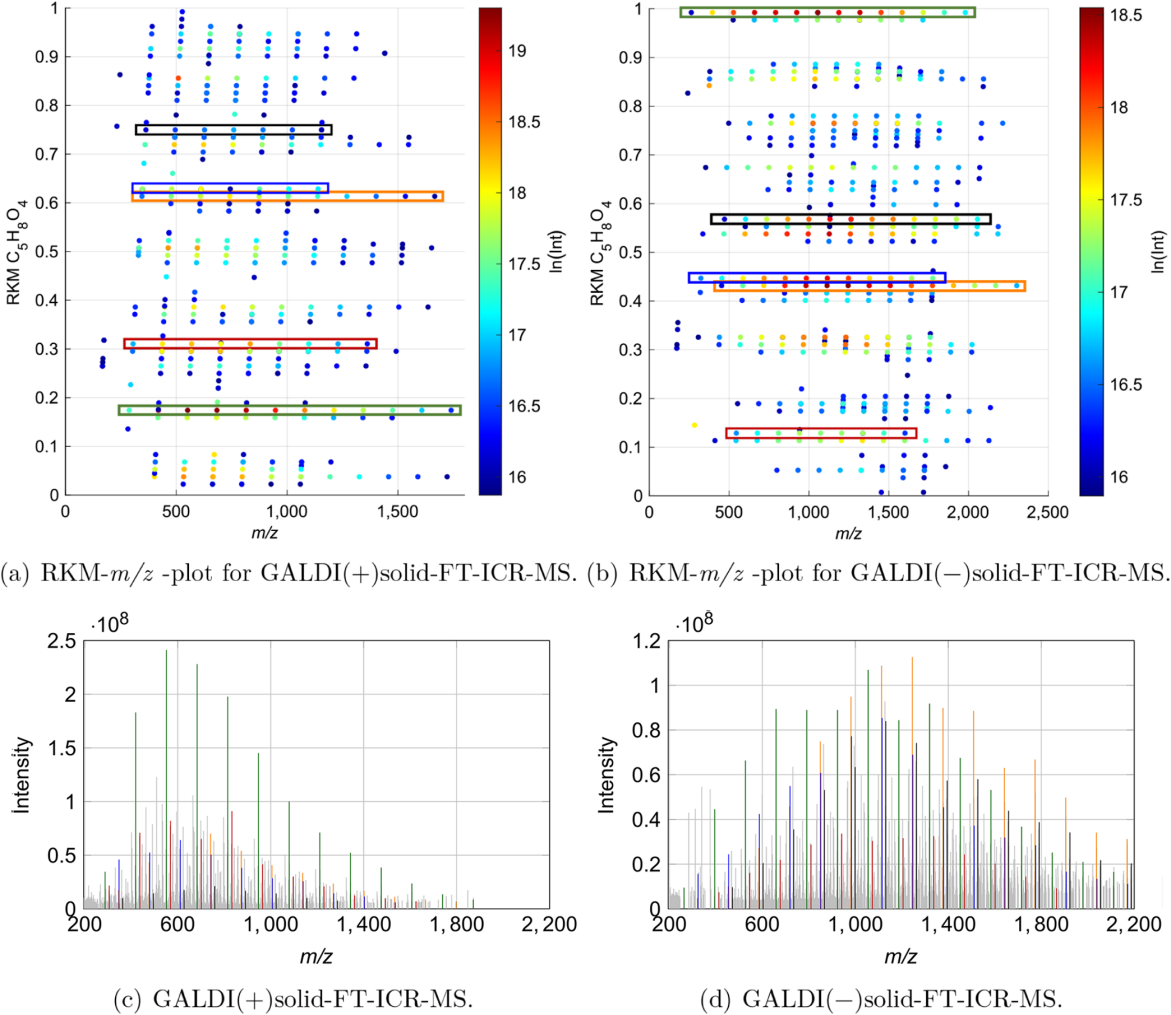
RKM-*m*/*z* plots and mass spectra for both ionization modes for the solid-state analysis routines are presented. The observed intensities of the data points in (a) and (b) are presented logarithmic and color-coded (blue: low intensity; green to yellow: medium intensity; red: high intensity). The colors of the signals in (c) and (d) as well as the associated markings in (a) and (b) correspond to the following oligomer series: red = [X_n_], green = [X_n_−H_2_O], blue = [X_n_Ac], orange = [X_n_(MeGlcA)–H_2_O], and black = [X_n_(MeGlcA)]; where X corresponds to xylose, Ac to acetylated species, and MeGlcA to 4-O-methylglucuronic acid.

**Table 3:** Summary of the Selected Oligomer Series for the Negative and Positive Ionization Modes for the Solid-State Analysis Routines.^a^

ionization mode	*m*/*z* range	structural proposal
negative	413.130076 to 1,865.595661	[X_n_]
	263.077241 to 2,111.670149	[X_n_−H_2_O]
	323.098370 to 2,171.692037	[X_n_Ac]
	321.082710 to 2,301.717109	[X_n_(MeGlcA)−H_2_O]
	471.135592 to 2,187.685963	[X_n_(MeGlcA)]
positive	305.084302 to 1,625.507601	[X_n_]
	287.073745 to 1,739.539127	[X_n_−H_2_O]
	347.094864 to 1,535.473746	[X_n_Ac]
	345.079225 to 1,797.545210	[X_n_(MeGlcA)−H_2_O]
	363.089766 to 1,287.385642	[X_n_(MeGlcA)]

a In this, X corresponds to xylose, Ac to acetylated species, and MeGlcA to 4-O methylglucuronic acid.

As presented in Table 3, the selected oligomer series exhibit a range of structural features, including an acidic substitution with 4-O-methylglucuronic acid or an acetylation. In addition, there are series that do not exhibit substitution. Furthermore, it is evident that during ionization, a dehydration process occurs, resulting in the formation of additional oligomer series. As illustrated in Figures 6a and b, the RKM values of these series are distinctly different, allowing for their clear differentiation.

As previously mentioned, there is a variance in intensity between the two ionization modes, with the positive mode demonstrating an overall higher signal intensity. However, upon further examination, it becomes evident that this observation is not universally applicable to all signals. As demonstrated in Figures 6c and d, the signal intensity of the unsubstituted series ([X_n_] and [X_n_−H_2_O]) increases significantly in positive ionization mode, while the signal intensity of the acidic substituted series ([X_n_(MeGlcA)] and [X_n_(MeGlcA)–H_2_O]) decreases. Conversely, the acetylated series ([X_n_Ac]) displays a minimal response to alterations in ionization modes. These observations indicate that ionization efficiency varies not only by ionization mode but also by the structural characteristics of the analyzed molecules. In accordance with these findings, it is strongly recommended to employ both ionization modes for a comprehensive characterization of xylans.

### IR and NMR Analyses

For cross-verification of the MS results, IR and solid-state NMR analyses were conducted. These data should be utilized to assess whether the observed MS results are accurate and to support the structural characterization of the beechwood xylan sample.

The IR spectrum (Figure 7) thereby contains bands that are characteristic of polysaccharides. These include the stretching vibrations of hydroxy groups (ν­(O–H)) at 3,342.17 cm^–1^, the C–H-stretching vibrations at 2,871.61 cm^–1^ and the C_1_–H deformation vibration at 894.85 cm^–1^, which is typical for β-1,4-glycosidic bonds.^41,42^

**Figure 7:**
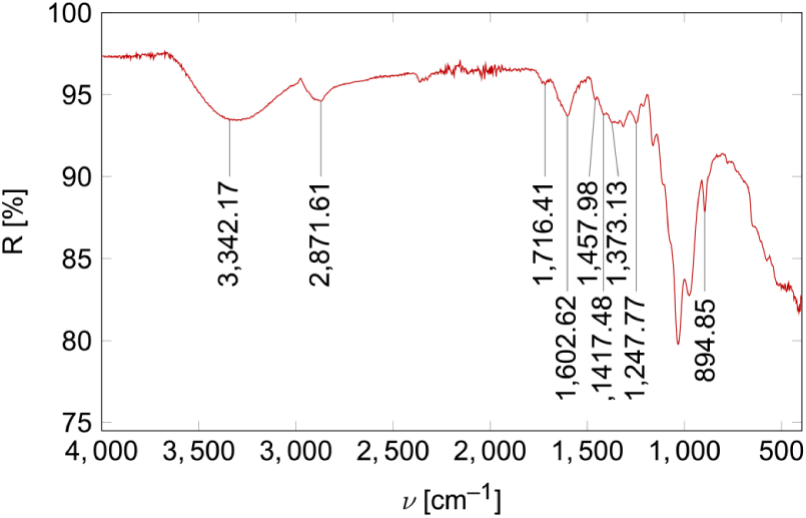
ATR-FT-IR spectrum of the powdered beechwood xylan sample.

The results also indicate an acidic substitution of the xylose main chain, with bands at 1,602.62 cm^–1^ and 1,417.48 cm^–1^, which are typical for the asymmetric and symmetric COO^–^ stretching vibrations in 4-O-methylglucuronic acid.^42^ Also, the bands at 1,750 to 1,700 cm^–1^ (ν­(C=O) in acids or esters)^41^ and 1,247.77 cm^–1^ (ν­(C–O) in acids)^43^ support this conjecture. It is important to note that the signal around 1,600 cm^–1^ could probably be overlapped by the H–O–H deformation band of water.^44^ The bands at 1,457.98 cm^–1^ and 1,373.13 cm^–1^ are associated with the asymmetric and symmetric deformation vibrations of methyl groups (δ_as/s_(CH_3_)).^42^ This is consistent with the proposed substitution of the main chain with MeGlcA. Furthermore, it may also indicate a partial acetylation of the main chain, which is a common occurrence in xylans originating from dicots, such as beeches.^3^

In addition to the IR analysis, a ^13^C solid-state NMR analysis (see Figure 8) was conducted to obtain further structural insights into the sample. The results of the NMR analysis thereby align with the observations made in the IR spectrum.

**Figure 8:**
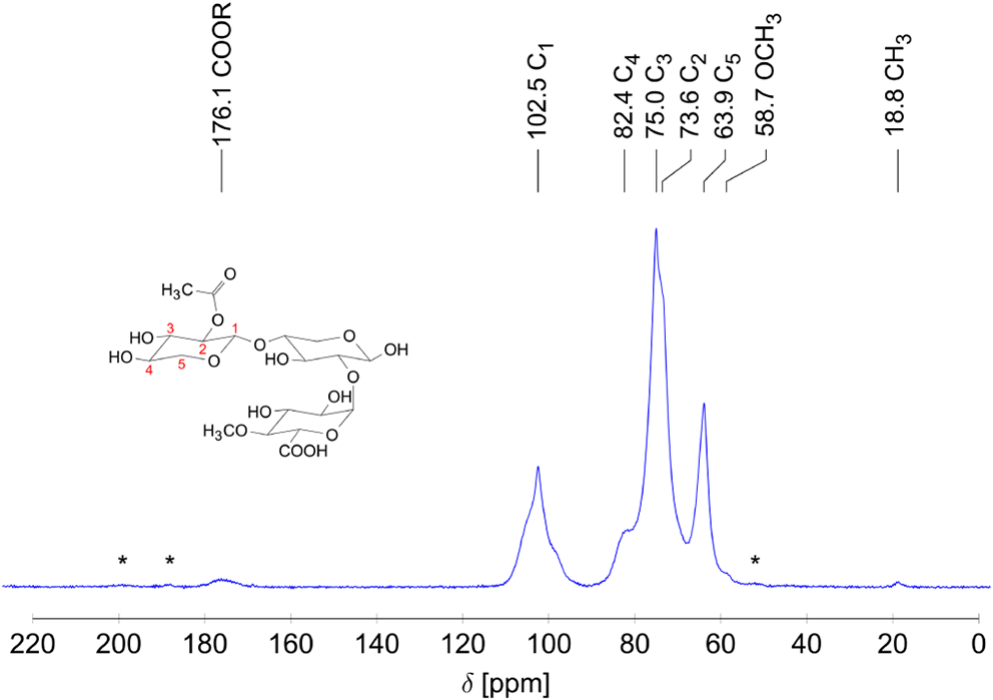
^13^C–CP/MAS NMR spectrum of the beechwood xylan including an exemplary structure of the expected possible substitutions on the xylose chain. The NMR spectrum was recorded on a 400 MHz spectrometer. The asterisks (*) mark the spinning sidebands.

The NMR spectrum shows signals corresponding to the five ring carbon atoms of the xylose monomers (see exemplary structure in Figure 8) at chemical shifts of 102.5 ppm (C_1_), 82.4 ppm (C_4_), 75.0 ppm (C_3_), 73.6 ppm (C_2_) and 63.9 ppm (C_5_), with the assignment based on the study by Dinand et al.^45^ Additionally, the broad signal in the chemical shift range of 182.0 to 167.0 ppm could be associated with the C=O group, which occurs in the carboxyl group of the MeGlcA^45^ and in the ester group of an acetyl group.^46^ The presence of both substituents is corroborated by the signals at 58.7 and 18.8 ppm, which can be attributed to methoxy and methyl groups. The former is likely to correspond to the methoxy group in MeGlcA, as referenced by Dinand et al.^45^ In contrast, the latter probably is associated with the acetyl groups on the main chain of the polymer, as documented in the study by Duan et al.^46^ Given the small size of this signal, it can be assumed that the degree of acetylation is low and likely less than 5%. However, it is important to note that the NMR spectrum does not provide quantitative data. Consequently, this estimate is considered approximate.

Despite the strong alignment between the various analytical approaches, a notable difference is observed between the results of NMR/IR spectroscopy and the mass spectrometric data. In the former spectra, no indications are found for the presence of additional compound classes, while the latter (see Figure 5) clearly reveal the presence of other compound classes beyond carbohydrates in the sample. These are not observed in the NMR and IR spectra due to their low concentrations within the sample. This makes them undetectable in the solid-state NMR spectrum and causes them to be overlaid by the polysaccharide vibrations in the IR spectrum. These compound classes are characterized by an aromatic nature, which leads to a high ionizability and, consequently, an enhanced detectability in mass spectrometry, despite their low concentrations, leading to their visibility in the mass spectra.

In summary, the results of the IR and NMR analyses align with the mass spectrometric results and help us to gain deeper insight into the sample.

## Conclusion

The utilization of biopolymers, such as xylans, as a regenerative alternative to fossil resources requires the determination and differentiation of the structure of these polymers from different sources. The development of analysis methods that facilitate a rapid and comprehensive overview of the structural composition of the biopolymer in question is therefore essential. Thus, the objective of this study was to develop novel and efficient preparation and analysis methods for the characterization of hemicelluloses using ultrahigh-resolving mass spectrometry. Utilizing GALDI-FT-ICR-MS, four methods were developed to characterize the soluble and insoluble compounds in a beechwood xylan sample in the positive and negative ionization modes. A comprehensive data evaluation revealed that employing positive and negative ionization modes is essential for a thorough characterization of xylans. Furthermore, it was demonstrated that a preliminary extraction of soluble xylan molecules is unnecessary, as a direct solid-state analysis can adequately depict the sample composition. Additional IR and NMR analyses confirmed the validity of the mass spectrometric results. Additionally, the analyses contributed to the identification of the sample as a partially acetylated glucuronoxylan (acetylation degree approximately <5%) due to a substitution with 4-O-methylglucuronic acid, which is typical for hardwoods, like beech.^4^ The HRMS analyses also enabled the detection of a structurally distinct homologous series of xylan compounds.

A more detailed insight into the sample structure can be achieved using tandem-MS analyses. Moreover, the application of the developed methods to other xylan or hemicellulose samples from different sources is important to further verify the analysis routines and demonstrate their versatility across diverse sample systems. A future publication will present the outcomes of the research conducted based on the work presented here. The developed analysis routines thereby present a fast and efficient way to structurally characterize xylans. The additional structural knowledge that can be obtained using these methods may help to develop more effective isolation, purification, and derivatization procedures for these polysaccharides. This will hopefully lead to more efficient and versatile utilization of xylans and other hemicelluloses in the future.

## Supplementary Material



## Notes

More detailed information on the GALDI-MS method development and optimization process and on the RSD values for the total ion current, alongside statistical bar chart plots about the oxygen-containing compound classes, n_C_–DBE plots for all four developed methods and van Krevelen plots as well as RKM-*m*/*z* plots and mass spectra of selected oligomer series for the liquid-state analysis routines (PDF)
