# Supplementary material for: Development of GALDI-FT-ICR-MS Methods for the Analysis of Xylan Oligomers
Source: Biomacromolecules. 2025 Oct 28;26(11):7897–908. doi: 10.1021/acs.biomac.5c01386 (PMC12606643; doi:10.1021/acs.biomac.5c01386)
Supplement: Supplementary file 1 [file bm5c01386_si_001.pdf]

SUPPORTING INFORMATION

Development of GALDI-FT-ICR-MS Methods for the Analysis of Xylan Oligomers

Klara Sander, Jan Zuber, Erica Brendler and Carla Vogt

Institute of Analytical Chemistry, TU Bergakademie Freiberg, Lessingstrasse 45, Freiberg, 09599, Germany

E-Mail: Klara.Sander@chemie.tu-freiberg.de; Jan.Zuber@chemie.tu-freiberg.de

Contents

S1 Method development and optimization of the solid- and liquid-state GALDI(+/−)-FT-ICR-MS analysis routines 4

S2 Relative standard deviation for the total ion current (TIC) 16

S3 Statistical evaluation of the oxygen containing heteroatomic classes for the solid- and liquid-state GALDI(+/−)-FT-ICR-MS methods 17

S4 Van Krevelen plots of the liquid-state GALDI(+/−)-FT-ICR-MS methods and the van Krevelen based relative overall composition for all methods 19

S5 n_C-DBE plots for the solid-state analysis routines 21

S6 Oligomer series labeled mass spectra of the solid-state GALDI-FT-ICR-MS methods in both ionization modes 22

S7 n_C-DBE and RKM-*m/z* plots of the liquid-state GALDI(+/−)-FT-ICR-MS methods 23

List of Figures

S1 Comparison of the mass spectrometric results for the optimization of the applied solvent for the xylan extraction. 4

S2 Comparison of the mass spectrometric results for the optimization of the applied H_2O/DMSO ratio of the solvent for the xylan extraction. 5

S3 Comparison of the mass spectrometric results for the optimization of the extraction temperature T and duration t for pure DMSO (blue) and the solvent mixture with a H_2O/DMSO ratio of 25:75 (orange) as solvents for the xylan extraction. 6

S4 Comparison of the mass spectrometric results for the optimization of the extraction concentration (c) for the xylan extraction. 7
S5 Comparison of the mass spectrometric results for the optimization of the applied amount of graphite (m_g) for the liquid-state GALDI-MS analyses in the positive (orange) and negative ionization mode (blue). 8
S6 Comparison of the mass spectrometric results for the optimization of the applied ionization supplement for the liquid-state GALDI-MS analyses in the positive and negative ionization mode. 9
S7 Comparison of the mass spectrometric results for the optimization of the concentration of the ionization supplement for the liquid-state GALDI-MS analyses for both ionization modes. 9
S8 Comparison of the peak number for the optimization of the TOF for the liquid-state GALDI-MS analyses in the positive (orange) and negative ionization mode (blue). Thereby the TOF values were varied in a range of 0.7 to 2.2 ms for the negative ionization mode and 0.7 to 1.8 ms for the positive ionization mode. 10
S9 Averaged and blank corrected mass spectra of the beechwood xylan sample obtained under varying S/G ratios (LDI, 10:1, 1:1, and 1:10 (w/w)) in the negative ionization mode. 12
S10 Comparison of the mass spectrometric results for the optimization of the S/G ratio for the negative ionization mode. 13
S11 Comparison of the mass spectrometric results for the optimization of the total amount of solids (m_t) for the positive ionization mode. 13
S12 Comparison of the mass spectrometric results for the optimization of the co-solvent for the positive ionization mode. 14
S13 Comparison of the mass spectrometric results for the optimization of the applied $CHCl_3$/DMSO ratio for the positive ionization mode. 14
S14 Statistical overview of the molecular formula evaluation for the four developed analysis methods, illustrating the total number of molecular formulae for selected oxygen containing classes between O_2 and O_{70}. 17
S15 Statistical overview of the molecular formula evaluation for the four developed analysis methods, illustrating the relative abundance of molecular formulae for selected oxygen containing classes between O_2 and O_{70}. 18
S16 van Krevelen plots of the xylan sample analyzed using liquid-state GALDI-FT-ICR-MS. The observed intensities are presented logarithmic and color-coded (blue: low intensity, green to yellow: medium intensity, red: high intensity).The boundaries for the compound class regions are defined according to Ayala-Ortiz et al.[1] 19
S17 Graphical representation of the percentage of assigned molecular formulae in the different compound classes based on the van Krevelen data for all four developed methods. 20
S18 n_C-DBE-n_O plots for the solid-state GALDI-FT-ICR-MS for the positive and negative ionization mode. The number of oxygen atoms in each molecule is visualized by a color bar ranging from 0 to 70 for both ionization modes. 21
S19 Mass spectra for both ionization modes for the solid-state analysis routines are presented. The colors of the signals correspond to the following oligomer series: red = $[X_n]$, green = $[X_n - H_2O]$, blue = $[X_nAc]$, orange = $[X_n(MeGlcA) - H_2O]$, and black = $[X_n(MeGlcA)]$; where X corresponds to xylose, Ac to acetylated species and MeGlcA to 4-O-methylglucuronic acid. 22
S20 n_C-DBE-n_O plots for the liquid-state GALDI-FT-ICR-MS for the positive and negative ionization mode. The number of oxygen atoms in each molecule is visualized by a color bar ranging from 0 to 60 for both ionization modes. 23

S21 RKM-m/z plots for both ionization modes for the liquid-state analysis routines are presented. The observed intensities of the data points are presented logarithmic and color-coded (blue: low intensity, green to yellow: medium intensity, red: high intensity) The markings correspond to the following oligomer series: red = $[X_n]$, green = $[X_n - H_2O]$, blue = $[X_nAc]$, orange = $[X_n(MeGlcA) - H_2O]$, and black = $[X_n(MeGlcA)]$; where X corresponds to xylose, Ac to acetylated species and MeGlcA to 4-O-methylglucuronic acid. 24
S22 Mass spectra for both ionization modes for the liquid-state analysis routines are presented. The colors of the signals correspond to the following oligomer series: red = $[X_n]$, green = $[X_n - H_2O]$, blue = $[X_nAc]$, orange = $[X_n(MeGlcA) - H_2O]$, and black = $[X_n(MeGlcA)]$; where X corresponds to xylose, Ac to acetylated species and MeGlcA to 4-O-methylglucuronic acid. 25

List of Tables

S1 Summary of the values for the relative standard deviation (RSD) of the total ion current (TIC) for the four developed methods, determined via two different approaches. 16
S2 Percentage of assigned molecular formulae in the different compound classes based on the van Krevelen data for all four developed methods. 19
S3 Summary of the selected oligomer series for the negative and positive ionization mode for the liquid-state analysis routines. In this X corresponds to xylose, Ac to acetylated species and MeGlcA to 4-O-methylglucuronic acid. 23

S1 Method development and optimization of the solid- and liquid-state GALDI(+/−)-FT-ICR-MS analysis routines

During method development, the criteria employed for selecting the most suitable options for sample preparation and ionization parameters generally included, in addition to the spectral appearance, the values for peak number, total ion current (TIC) or mean m/z. The peak number thereby describes the number of signals that exhibit a S/N ratio of at least 5. All of these parameters were determined from the blank corrected data sets. The method development process started with developing preparation and analysis routines for the liquid-state GALDI-MS in the positive and negative ionization modes. Therefore, the soluble xylan molecules were extracted using an ultrasonic bath. In the initial phase of method development, the extraction process was optimized with respect to the extraction solvent. Accordingly, seven different solvents were examined, covering a wide range of polarity: sodium hydroxide (NaOH, 5 %),water (H_2O), isopropanol (i-PrOH), dimethyl sulfoxide (DMSO), chloroform ($CHCl_3$), toluene and n-hexane. As demonstrated in Figures S1(a) and S1(b) the results reveal that the highest number of peaks and the highest TIC values were obtained when using NaOH, water and DMSO as extraction solvents. Furthermore, the mass spectra revealed that, with exception of water and DMSO, there was an absence of sample signals in the mass spectra for the other solvents under investigation.

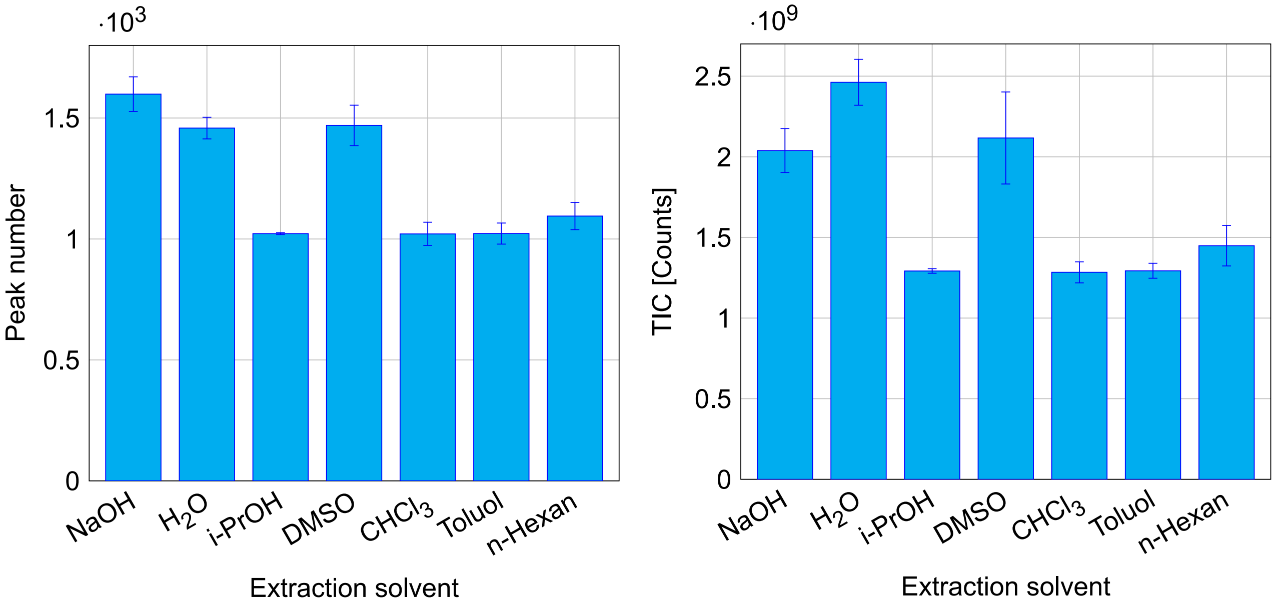

(a) Peak number

(b) TIC

Figure S1: Comparison of the mass spectrometric results for the optimization of the applied solvent for the xylan extraction.

Given the fact that the peak numbers and TIC values for water and DMSO were in a similar range, it was investigated whether a solvent mixture of water and DMSO would improve the analytical results. Therefore, the five H_2O/DMSO ratios of 100:0, 75:25, 50:50, 25:75 and 0:100 (v/v) were analyzed. The results of the aforementioned experiments, with respect to the peak number and TIC, are illustrated in Figures S2(a) and S2(b). The findings indicate that the H_2O/DMSO ratios of 25:75 and 0:100 (v/v) are suitable for the analysis of the xylan sample. Consequently, it was determined that the subsequent optimization step would be conducted on both preparation variants, with the objective of optimizing the extraction duration and temperature.

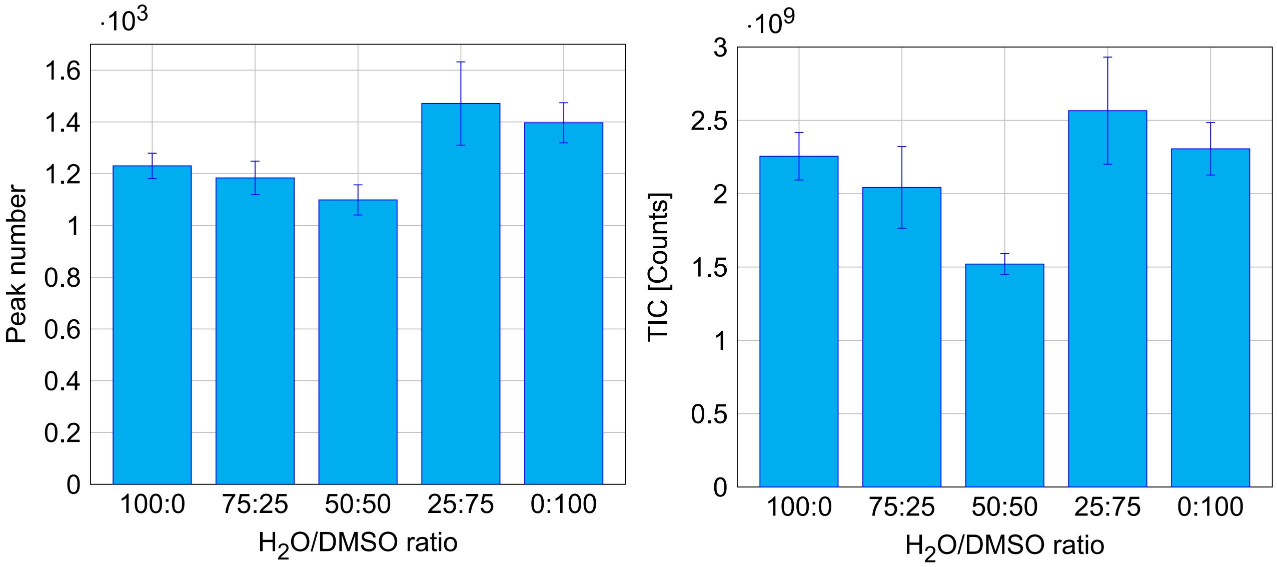

(a) Peak number.

(b) TIC.

Figure S2: Comparison of the mass spectrometric results for the optimization of the applied H_2O/DMSO ratio of the solvent for the xylan extraction.

The extraction was, therefore, carried out at temperatures of 30 °C, 40 °C and 50 °C for durations of 5 min, 15 min and 25 min, respectively. The findings demonstrated that the optimal extraction conditions for pure DMSO as the solvent were determined to be an extraction temperature of 30 °C and a duration of 15 min. This extraction method resulted in the highest peak number and TIC, as illustrated in Figures S3(a) and S3(b). Conversely, the solvent mixture exhibited no distinct optimum (see Figures S3(a) and S3(b)). Additionally the results for pure DMSO were, in nearly all cases, superior to those of the solvent mixture. Therefore, it was decided that pure DMSO should be utilized as extraction solvent. Consequently, the extraction temperature was set to 30 °C and the extraction duration was determined to be 15 min for all forthcoming experiments.

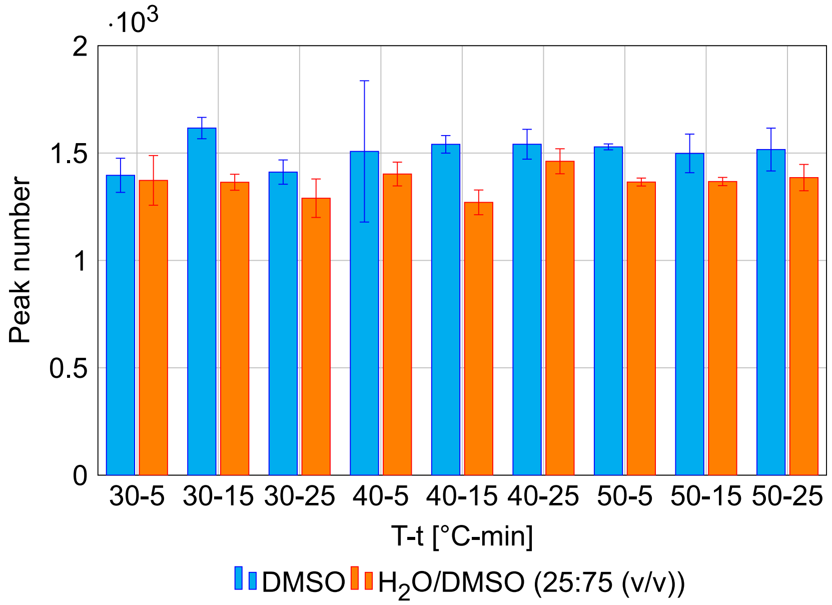

(a) Peak number.

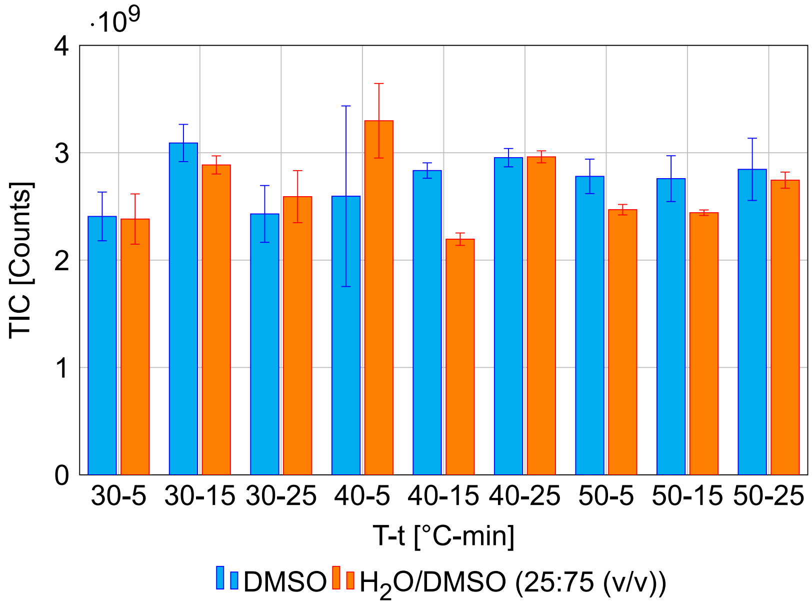

(b) TIC.

Figure S3: Comparison of the mass spectrometric results for the optimization of the extraction temperature T and duration t for pure DMSO (blue) and the solvent mixture with a H_2O/DMSO ratio of 25:75 (orange) as solvents for the xylan extraction.

In the final stage of the optimization process for the xylan extraction, the extraction concentration was adjusted. Therefore, the five xylan concentrations of 1 g/L, 5 g/L, 10 g/L, 15 g/L and 20 g/L were examined. The findings indicate that a xylan concentration of 10 g/L is best suited for the extraction process, as demonstrated by the peak number and TIC results (see Figures S4(a) and S4(b)).

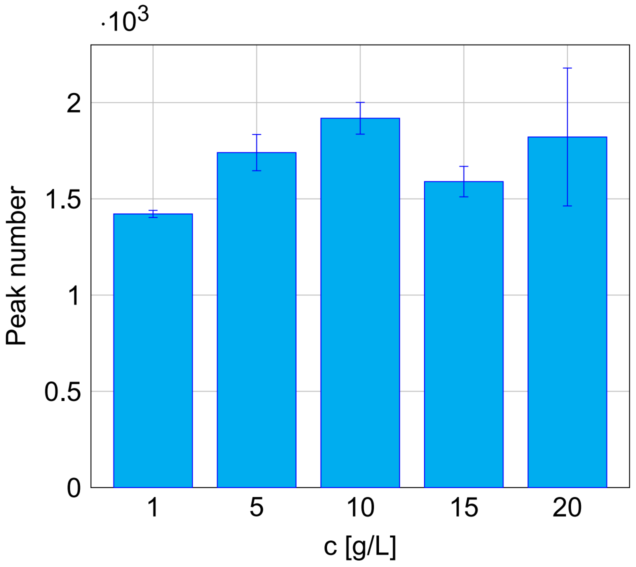

(a) Peak number.

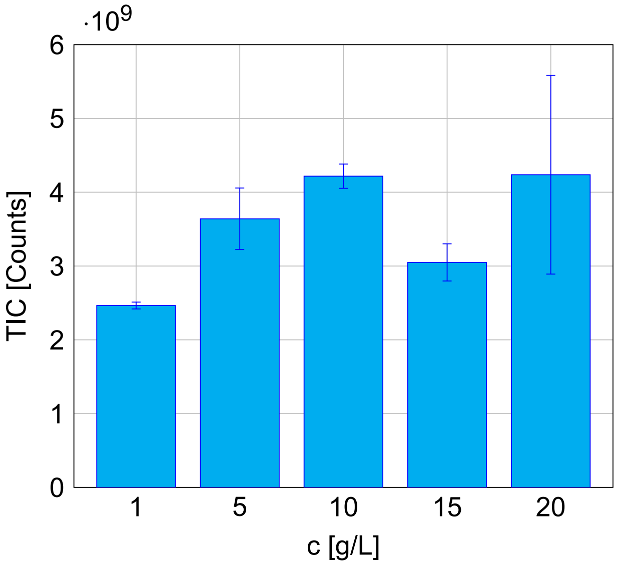

(b) TIC.

Figure S4: Comparison of the mass spectrometric results for the optimization of the extraction concentration (c) for the xylan extraction.

In the next step of method development, the GALDI sample preparation was optimized for both ionization modes. The sample preparation procedure thereby includes the suspension of high purity graphite in the xylan extract and an additional solvent (co-solvent). The suspension is then homogenized in an ultrasonic bath for 10 min and spotted onto a MALDI target. As co-solvents tetrahydrofuran (THF) was used for the negative ionization mode, while methanol (MeOH) was employed for the positive ionization mode. The co-solvent's function was on the one hand to further dilute the extract solution and on the other hand to enhance the volatility of the solvent mixture, a necessity arising from the low volatility of the DMSO. This low volatility of DMSO also necessitated the drying of the sample spots in a drying oven prior to measurement to ensure a relatively rapid evaporation of the solvents.

A significant factor in the optimization of the sample preparation is the amount of graphite (m_g) utilized in the formation of the sample suspension. In this regard, this parameter was optimized within a range of 0 mg to 50 mg in 10 mg steps. Thereby, it was determined that the absence of graphite resulted in an unsuccessful ionization of the xylan molecules. This phenomenon can be attributed to the lack of functional groups or ring systems capable of absorbing the laser radiation. Consequently, the molecules are not excited and thus no desorption and ionization take place. Conversely, a high excess of graphite (greater than 30 mg) has been observed to result in signal suppression, particularly for the positive ionization mode. This can be explained by the formation of a greater number of carbon clusters in the positive ionization mode, which are more readily ionized than the sample molecules. This trend is visualized in Figures S5(a) and S5(b), which present the peak number and TIC values in dependence of the graphite quantity. Thereby, it is evident that an optimum for both parameters is reached at 20 mg for the negative ionization mode, making it the most suitable for sample preparation in this mode. For the positive ionization mode the maximum TIC value is attained at an amount of graphite of 30 mg, while the peak number yields its highest values at 20 mg. Given the observation that the standard deviation for an amount of graphite of 30 mg is considerably smaller than for 20 mg, it was decided that 30 mg of graphite are more appropriate for the analysis of xylans in the positive ionization mode.

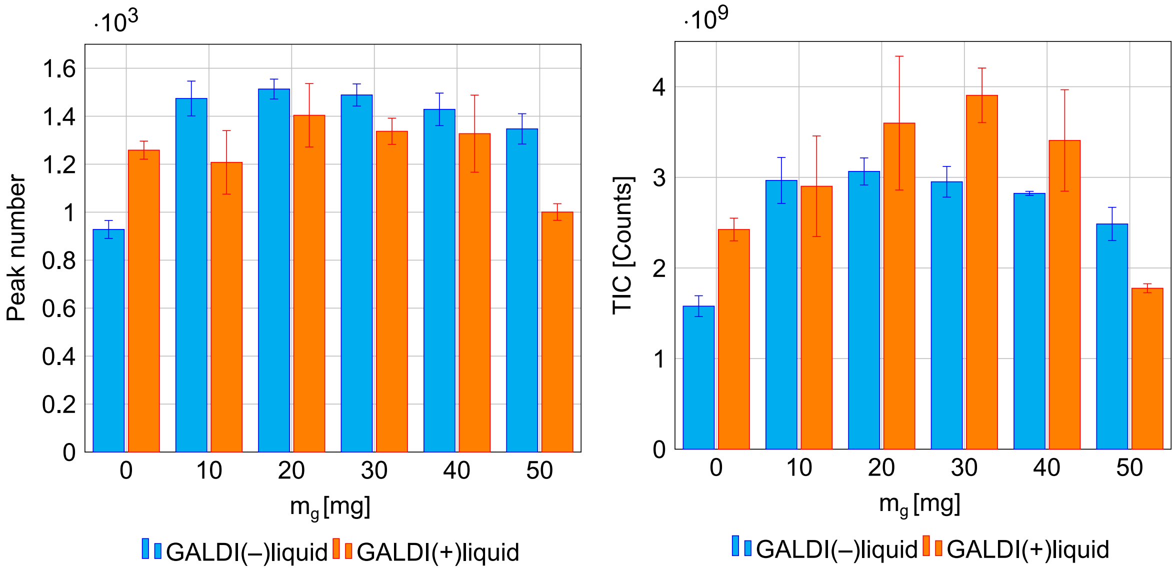

(a) Peak number. (b) TIC.

Figure S5: Comparison of the mass spectrometric results for the optimization of the applied amount of graphite (m_g) for the liquid-state GALDI-MS analyses in the positive (orange) and negative ionization mode (blue).

Additionally, the influence of ionization supplements was investigated, to support the formation of ions in the polysaccharide sample. In this regard, five different ionization supplements were tested in positive (sodium acetate (NaAc), ammonium acetate (NH_4Ac), sodium trifluoroacetate (NaTFA), ammonium trifluoroacetate (NH_4TFA) and trifluoroacetic acid (TFA)) and negative ionization mode (triethylamine (NEt_3), ammonia (NH_3), NaOH, calcium hydroxide ($Ca(OH)_2$) and sodium carbonate (Na_2CO_3)), respectively. Therefore, the supplements were added to the sample suspension in a concentration of 10 mM for both ionization modes. It was shown that the addition of bases for the negative ionization mode and organic salts in positive ionization mode indeed is beneficial for ionizing polysaccharide molecules. For the negative ionization mode the addition of NH_3 leads to the highest peak number and TIC values (see Figure S6(b)) and is therefore best suited for the analysis of xylan samples. For the positive ionization mode the results of the mass spectrometric measurements are presented in the Figure S6(a). Hereby it is visible that for NaTFA the highest TIC values are obtained. The peak number on the other hand shows higher values for NH_4Ac and NH_4TFA, indicating the formation of more different ion types for these two supplements. Based on the fact, that within the range of standard deviation the values nevertheless are quite comparable, the mass spectra also were similar and the main ion type formed during ionization is $[M + Na]^+$, it was decided to utilize NaTFA as ionization supplement in the positive ionization mode.

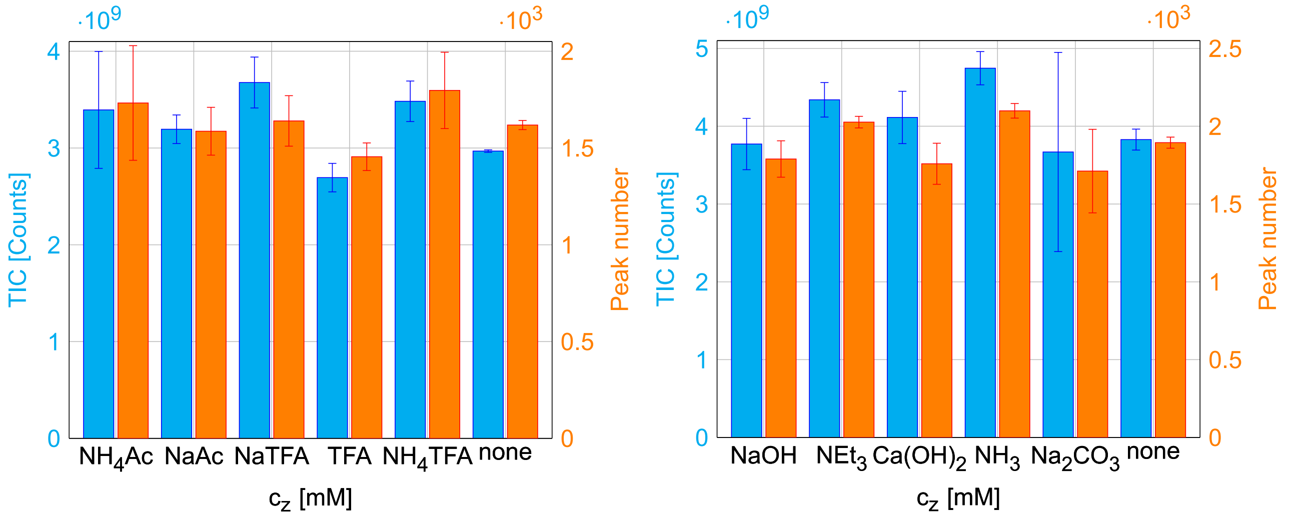

(a) TIC (blue) and peak number (orange) for GALDI(+)liquid. (b) TIC (blue) and peak number (orange) for GALDI(−)liquid.

Figure S6: Comparison of the mass spectrometric results for the optimization of the applied ionization supplement for the liquid-state GALDI-MS analyses in the positive and negative ionization mode.

Furthermore, the supplement concentration (c_z) was also optimized. Therefore the concentrations 1 mM, 5 mM, 10 mM, 50 mM and 100 mM were tested. Additionally, a sample without ionization supplement ($c_z = 0$ mM) was analyzed for comparison. Thereby it was decided that for the negative ionization mode a concentration of 50 mM is best suited for the analysis of xylans (see Figure 7(b)). Conversely, the decision regarding the positive ionization mode was more challenging. As demonstrated in Figure 7(a), the maximum values for the mean m/z are observed at 5 mM and 50 mM. Upon examination of the TIC, it becomes evident that the standard deviation is notably elevated for 50 mM. Consequently, this concentration is considered unfavorable for the analysis of xylans. It is evident that the TIC exhibits the highest value for 1 mM. A comparison between the data for 1 mM and 5 mM indicates that, despite the statistical data supporting 1 mM, the mass spectra are comparable and the sample preparation was preferable for 5 mM, which led to the decision to utilize 5 mM as the supplement concentration for the positive ionization mode.

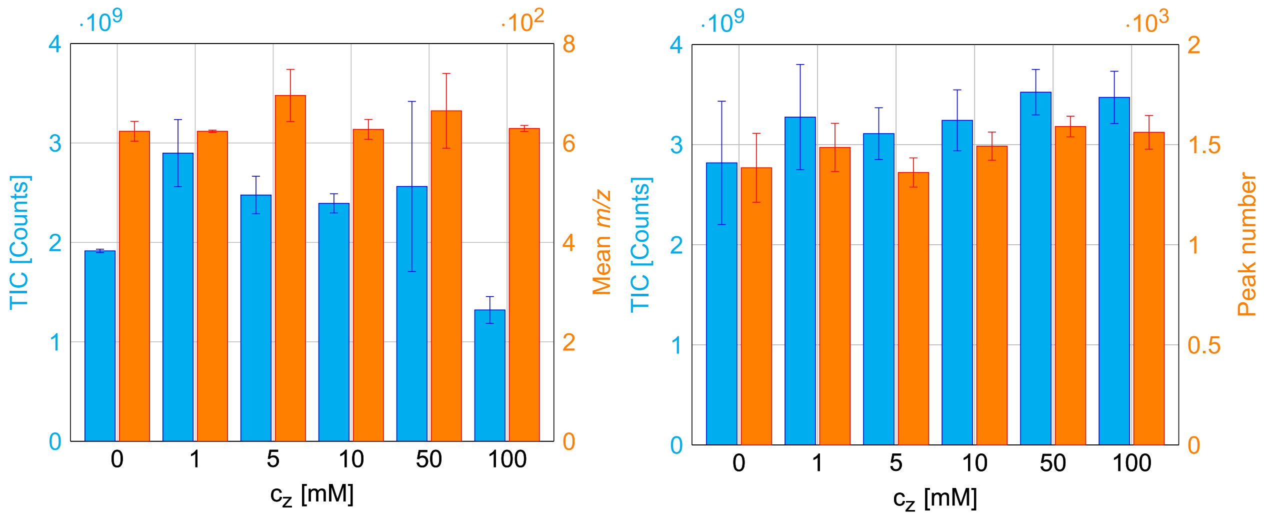

(a) TIC (blue) and mean m/z (orange) for GALDI(+)liquid. (b) TIC (blue) and peak number (orange) for GALDI(−)liquid.

Figure S7: Comparison of the mass spectrometric results for the optimization of the concentration of the ionization supplement for the liquid-state GALDI-MS analyses for both ionization modes.

To further enhance the spot quality, the potential of glycerol as an adhesive to fixate the suspension on the

target was examined. For the negative ionization mode the addition of glycerol indeed had a positive effect on the spot quality, whereas for the positive ionization mode, glycerol should not be added due to miscibility issues. The final step in the method development process involved optimizing the mass spectrometric parameters. Therefore, the time of flight (TOF) was optimized. The TOF describes the time between the ejection of the ions from the collision cell and their capture in the ICR-cell, thereby influencing the m/z range displayed in the mass spectrum. In order to ensure that the obtained mass spectra display the widest possible m/z range, the TOF was varied over a range of 0.7 to 2.2 ms for the negative ionization mode and 0.7 to 1.8 ms for the positive ionization mode with a step width of 0.1 ms. The TOF values were constrained to a more limited range for the positive ionization mode because the ions can not be separated and detected properly for TOF values exceeding 1.8 ms, due to the ion trap being overloaded. The decision regarding the optimal value was thereby influenced by additional features, including peak splitting, signal cancellation at lower m/z and the laser power required to form sample ions. Peak splitting is defined as the process of splitting a signal into two distinct signals due to a detector overload. This phenomenon can cause a number of issues, including the failure to detect blank value peaks during blank value correction or the incorrect assignment of m/z values during structural evaluation, resulting in an inaccurate data interpretation. Accordingly, the TOF that yielded the highest number of peaks (negative: TOF = 2.0 ms; positive: TOF = 1.6 ms), as illustrated in Figure S8, was not selected as the optimal solution. Instead, a reasonable compromise was reached and a TOF was selected, which resulted in a high number of peaks and the widest possible m/z range without signal cancellation or peak splitting. This compromise value was determined to be 1.3 ms for the negative ionization mode and 0.9 ms for the positive ionization mode. It is evident that the TOF value for the positive ionization mode is considerably lower than for the negative ionization mode. This is due to the fact that a higher quantity of ions is generated in the positive ionization mode, resulting in a more pronounced peak splitting even at relatively low TOF values.

All other mass spectrometric parameters were selected to be maintained at standard settings, as presented in Table 1 in the main part of the paper. Thereby, the laser power has to be optimized for each measurement to ensure high-quality results.

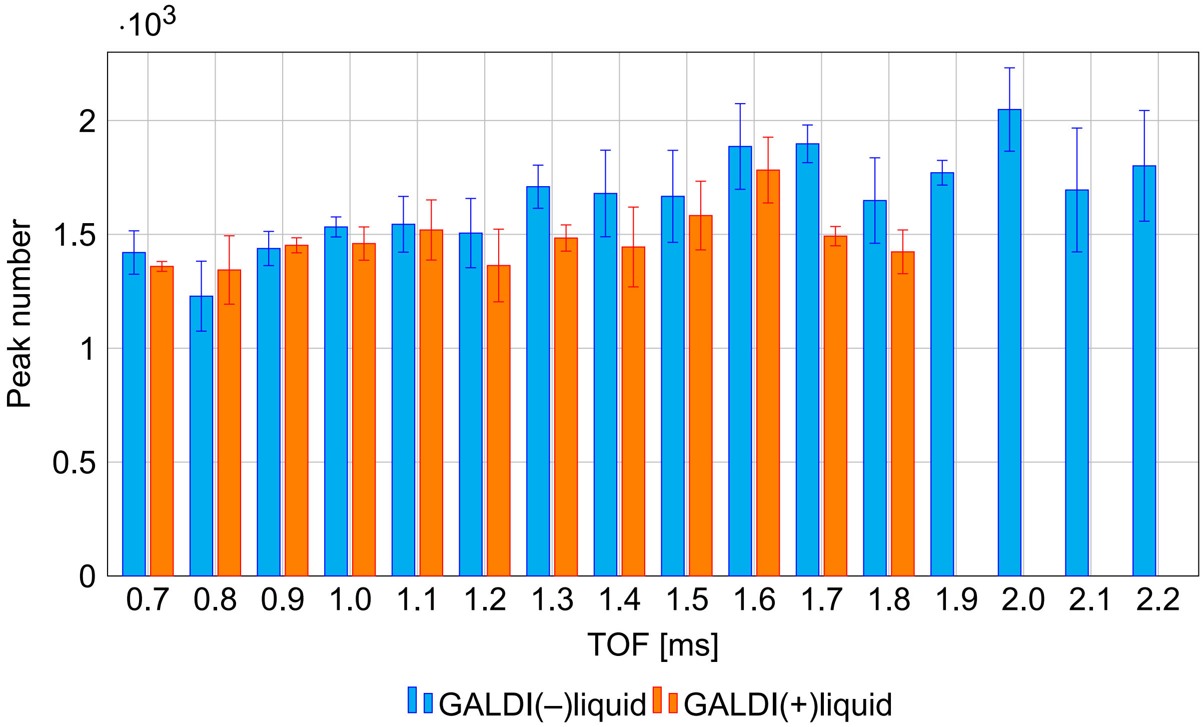

Figure S8: Comparison of the peak number for the optimization of the TOF for the liquid-state GALDI-MS analyses in the positive (orange) and negative ionization mode (blue). Thereby the TOF values were varied in a range of 0.7 to 2.2 ms for the negative ionization mode and 0.7 to 1.8 ms for the positive ionization mode.

For the method development of the solid-state GALDI(+/−)-FT-ICR-MS methods the powdered sample was directly mixed with the high purity graphite. The mixture was then suspended in a solvent, homogenized and the suspension was then spotted onto the MALDI target. The primary step of the method development process involved optimizing the sample-to-graphite ratio (S/G ratio). Therefore, the ratios 10:1, 5:1, 2:1, 1:1, 1:2, 1:5 and 1:10 (w/w, with a total solids content of 30 mg) were analyzed. Furthermore, the feasibility of ionizing the sample without the addition of graphite (LDI) was investigated. In Figure S10(a) the number of peaks versus the S/G ratio for the negative ionization mode is illustrated. Figure S10(b) shows the mean *m/z* as a function of the S/G ratio for the negative ionization mode (the data for the positive ionization mode are not shown here). It is evident that the generation of the most peaks and the largest molecules was achieved through LDI or a S/G ratio of 10:1. However it is also apparent that the standard deviation for these experiments is very high. Figure S9 additionally displays the mass spectra of three selected S/G-ratios and the LDI approach. It is evident that the mass spectra for the LDI and the high sample excess approach deviate significantly from those observed for a S/G ratio of 1:1 (w/w) and an elevated graphite excess. While the latter spectra display a typical xylan signal pattern, the former spectra show no sample signals at all. This can be attributed to the absence of graphite as a matrix (LDI), respectively the high sample excess (10:1 (w/w)), which result in negligible ionization of the xylan molecules, which is relatable to the absence of UV-absorbing structures in the xylan sample. Instead, the signals observed in these mass spectra, are most likely due to impurities and the graphite on the target. This results in the formation of carbon clusters that were not eliminated by the blank correction. In addition to the aforementioned findings, the signal intensity exhibits a maximum at a S/G ratio of 1:10 (w/w), as illustrated in Figure S9. The results, when considered with the relatively low standard deviation of the peak number, the comparable mean *m/z* to the other S/G ratios and the minimal required amount of sample, led to the conclusion that a S/G ratio of 1:10 (w/w) is the most appropriate for further investigations, which also applies to the positive ionization mode. This demonstrates that not only the statistical results are important for method optimization, but also the appearance of the mass spectra.

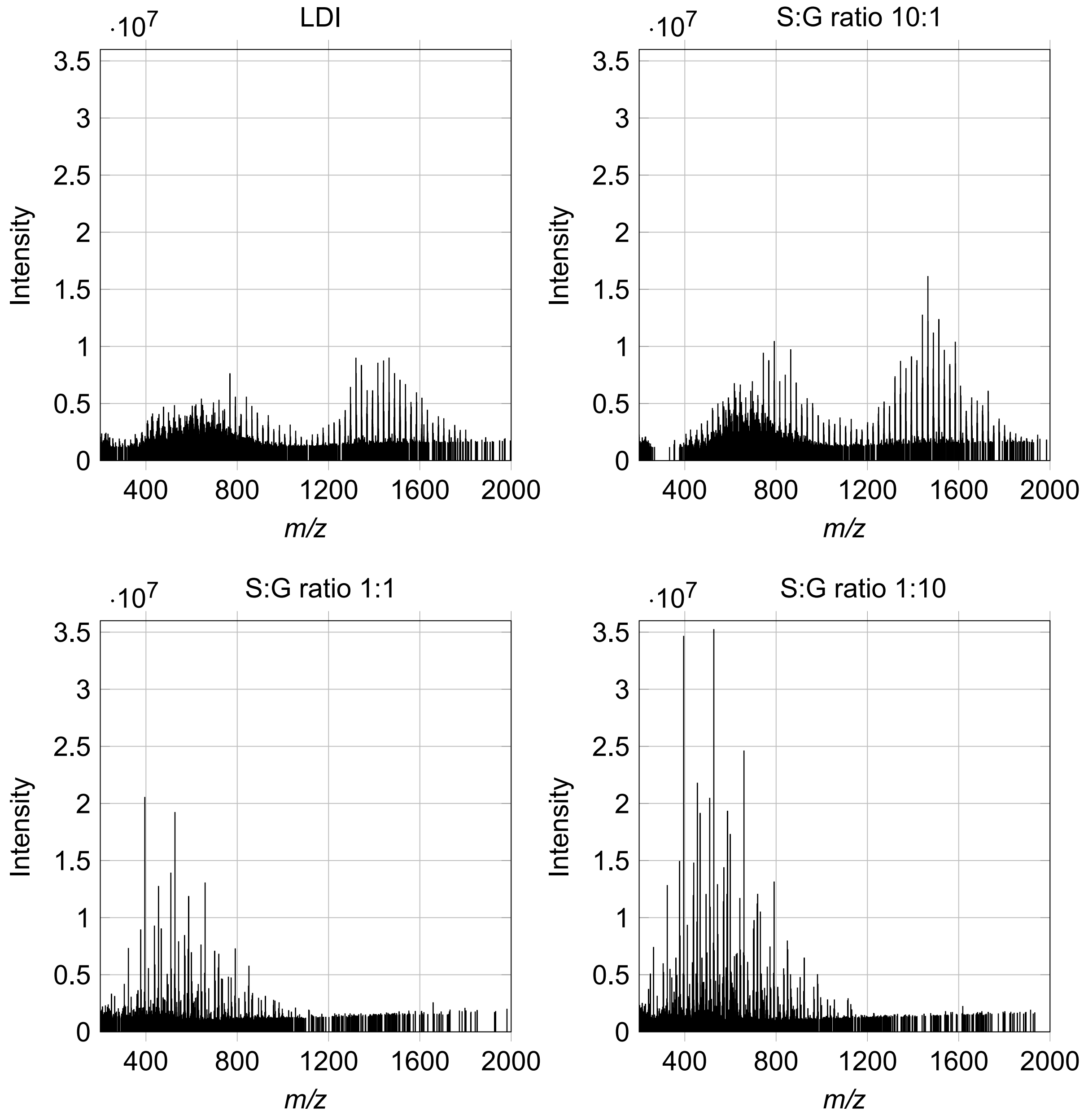

Figure S9: Averaged and blank corrected mass spectra of the beechwood xylan sample obtained under varying S/G ratios (LDI, 10:1, 1:1, and 1:10 (w/w)) in the negative ionization mode.

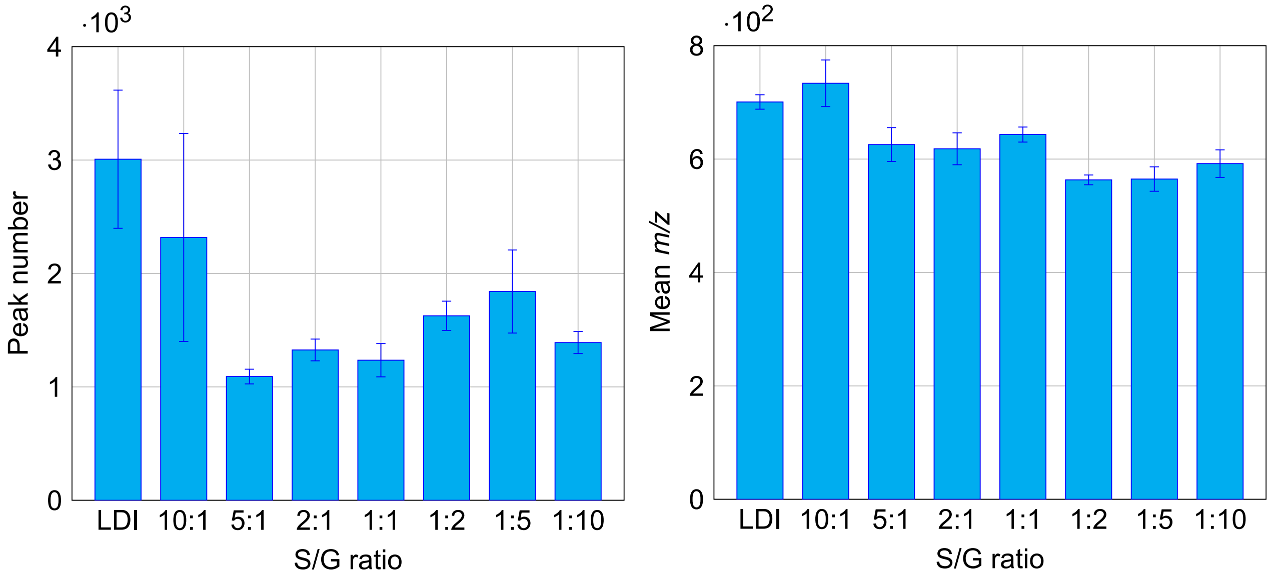

(a) Peak number. (b) Mean m/z .

Figure S10: Comparison of the mass spectrometric results for the optimization of the S/G ratio for the negative ionization mode.

Another important factor that impacts the spot quality is the total amount of solids (m_t) employed in the preparation of the sample suspension. It was determined that an optimization of this parameter was not necessary for the negative ionization mode (total amount of solids: 30 mg), as the quality of the sample spots was satisfactory. In contrast, for the positive ionization mode, the optimal results (see Figure S11) were achieved with a total solids content of 50 mg.

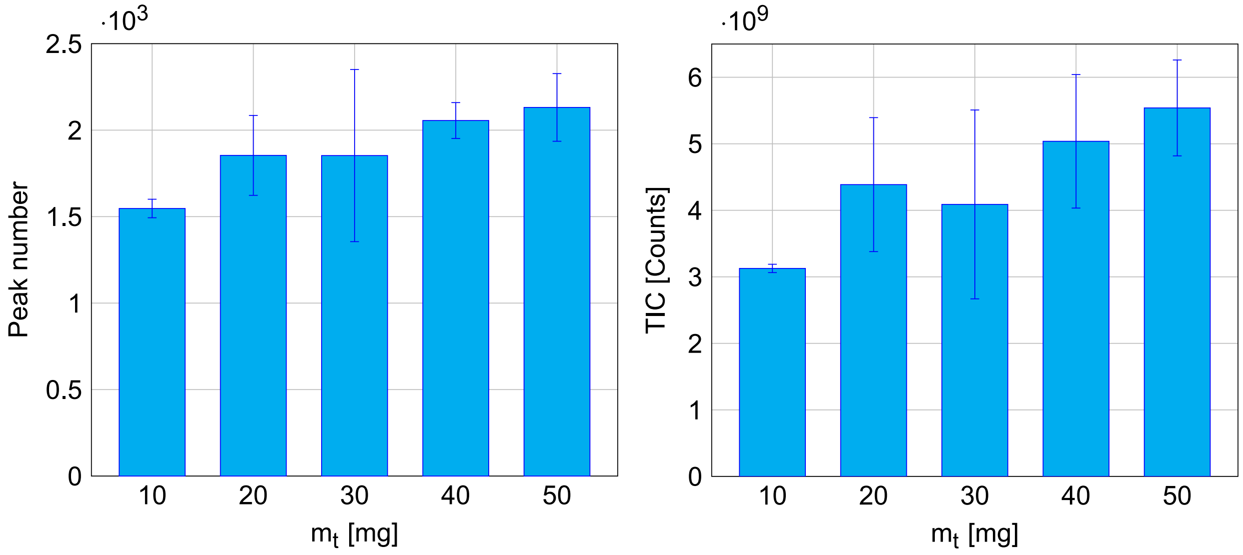

(a) Peak number. (b) TIC.

Figure S11: Comparison of the mass spectrometric results for the optimization of the total amount of solids (m_t) for the positive ionization mode.

As described in the main part of the paper, an approach was made to utilize the same solvents as for the liquid-state methods. This attempt proved to be successful only for the negative ionization mode (DMSO/THF (50:50 v/v)). In the positive ionization mode, it was necessary to further optimize the preparation method in terms of the co-solvent used in addition to DMSO. Therefore, the co-solvents H_2O, MeOH, i-PrOH, DMSO and $CHCl_3$ were examined. Thereby, the results obtained using $CHCl_3$ (see Figure S12) were found to be the most favorable.

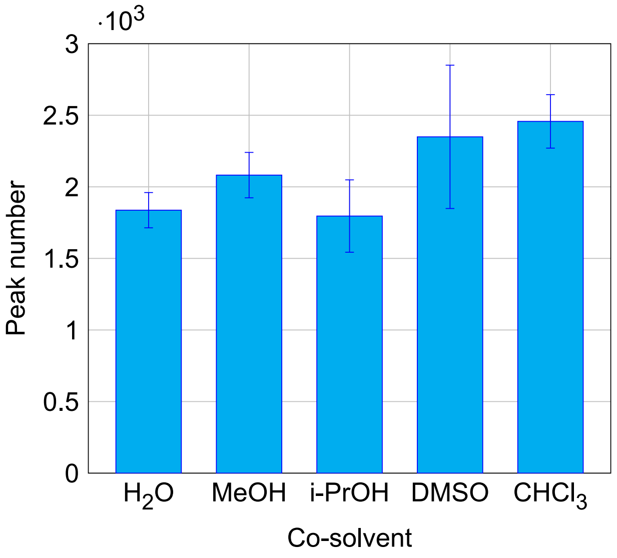

(a) Peak number.

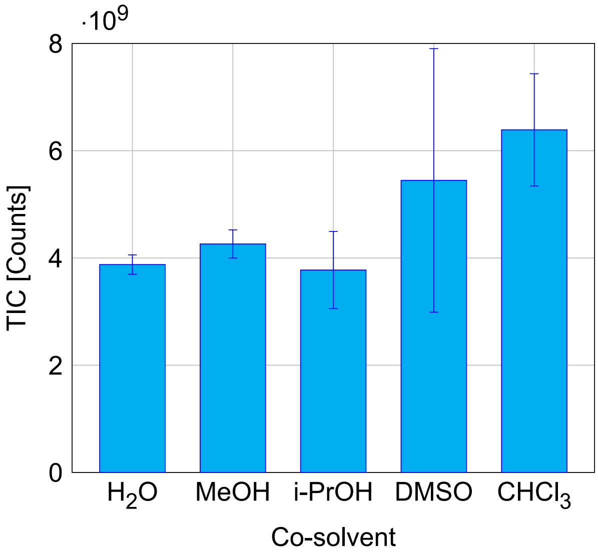

(b) TIC.

Figure S12: Comparison of the mass spectrometric results for the optimization of the co-solvent for the positive ionization mode.

Furthermore, the $CHCl_3$/DMSO ratio was optimized. Therefore, the $CHCl_3$/DMSO ratios of 50:50, 40:60, 30:70, 20:80, 10:90 and 0:100 (v/v) were investigated, as illustrated in Figure S13. It was determined that a ratio of 20:80 (v/v) of $CHCl_3$ and DMSO was the most suitable.

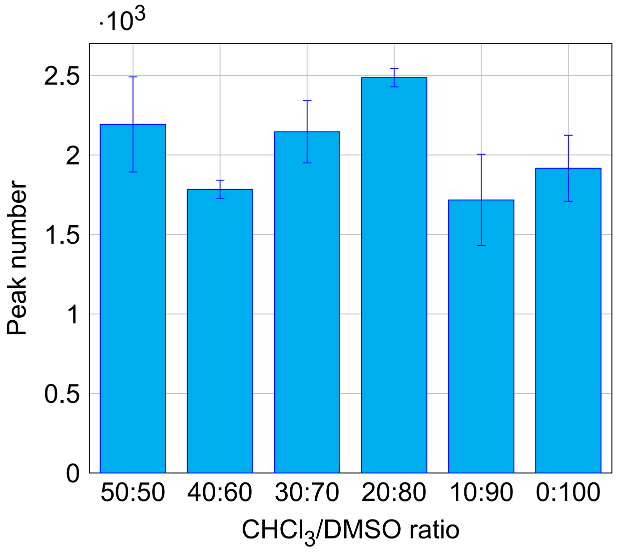

(a) Peak number.

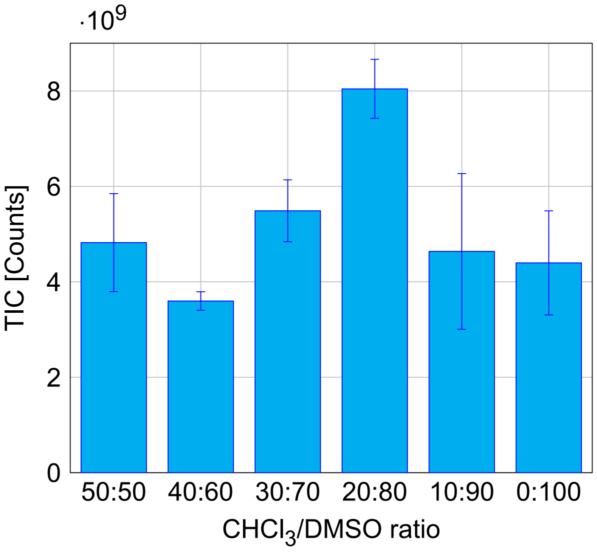

(b) TIC.

Figure S13: Comparison of the mass spectrometric results for the optimization of the applied $CHCl_3$/DMSO ratio for the positive ionization mode.

The remaining steps in the method development process were analogous to the solution analysis in terms of ionization supplements (optimum: negative: NH_3; positive: NH_4TFA) and their concentrations (optimum negative: 5 mM; positive: 50 mM) as well as the settings for the TOF (optimum negative: 1.4 ms, positive: 1.2 ms). In this case as well, the addition of glycerol has been demonstrated to further enhance the spot quality for the negative ionization mode; however, this approach has not been effective in positive ionization mode.

S2 Relative standard deviation for the total ion current (TIC)

Table S1 presents the relative standard deviation (RSD) for the TIC depending on different preparation approaches. Firstly, the spot-to-spot homogeneity was investigated by performing a triple determination on a single sample (described as ”1 preparation” in Table S1). Secondly, the reproducibility of the preparation methods was examined by performing three individual sample preparations and a single determination for each prepared sample (described as ”3 preparations” in Table S1). The TIC thereby quantifies the total amount of detected ions.

Table S1: Summary of the values for the relative standard deviation (RSD) of the total ion current (TIC) for the four developed methods, determined via two different approaches.

method	1 preparation	3 preparations
GALDI(−)solid	7.63 %	15.63 %
GALDI(−)liquid	6.28 %	11.97 %
GALDI(+)solid	3.95 %	7.59 %
GALDI(+)liquid	8.19 %	17.91 %

As demonstrated in Table S1, the RSD values for a single preparation are consistently below 10 % and are comparable for the negative ionization mode methods. For the positive ionization mode, the solid-state method demonstrates a RSD value that is approximately half of that obtained with the liquid-state method. This indicates, that in this case, the solid-state method exhibits a more uniform ionization.

For three separate preparations the RSD values double for all four methods. As already explained in the main part of the paper, this increase can be attributed to the higher influence of human factors on three separate sample preparations and the complexity of the sample system under examination. Polysaccharides demonstrate relatively low ionization efficiencies, resulting in irregular ionization and, consequently, variations among analyses. This has a substantial impact on the quantity of ions produced during ionization and hence the quantity of ions detected during analysis, thus resulting in elevated variability in the measured TIC values. The peak number and mean m/z values appear to be less affected, resulting for the most part in a more moderate or no increase in the RSD values for these parameters, as presented in the main part of the paper. However, when all the influencing factors are taken into account, RSD values below 20 % for the TIC also indicate a robust reproducibility of the developed analysis methods.

S3 Statistical evaluation of the oxygen containing heteroatomic classes for the solid- and liquid-state GALDI(+/−)-FT-ICR-MS methods

Figure S14 illustrates the total number of molecular formulae per oxygen containing class for all four analysis methods. A comparison of the solid- and liquid-state methods for each ionization mode reveals that, for the positive ionization mode, a higher number of molecular formulae is observed in the lower oxygen-containing classes (O_2 to O_{22}) for the GALDI(+)liquid method compared to the corresponding solid-state method. However, it is also evident that, for medium and higher oxygen numbers (O_{24} to O_{52}), the results are converging. For the negative ionization mode both analysis methods yield similar results for the lower and medium oxygen numbers (O_2 to O_{40}). Nevertheless, for the higher oxygen numbers (O_{42} to O_{70}) and thus the larger oligomers, the GALDI(−)solid method, as anticipated, leads to a greater number of molecular formulae than the liquid-state method. When comparing the ionization modes, it becomes apparent that the negative ionization mode results in the formation of larger molecules with medium to high oxygen numbers (O_{30} to O_{70}), while the positive ionization mode predominantly yields low to medium oxygen numbers (O_2 to O_{30}). This can be attributed to the differences in the TOF, as previously explained in the main part of the paper. Furthermore, the error bars illustrate that the ionization of the sample molecules is irregular, a phenomenon attributed to the molecule structure. Additionally the relative abundance of the heteroatomic classes is visualized in Figure S15. Herein, the oxygen-containing classes with the most significant molecular formulae can be identified. It is evident that the basic distribution of the data is analogous to that of the total number of molecular formulae. As expected, the oxygen-containing classes with a relatively low oxygen number are more abundant in the positive ionization mode (GALDI(+)liquid: O_8 to O_{18}; GALDI(+)solid: O_{10} to O_{20}), whereas medium oxygen numbers are most abundant for the negative ionization mode methods (GALDI(−)liquid: O_{22} to O_{36}; GALDI(−)solid: O_{30} to O_{40}). Thus in the negative ionization mode a higher number of larger oxygen containing oligomers is formed, whereas the positive ionization mode preferably leads to smaller oligomers.

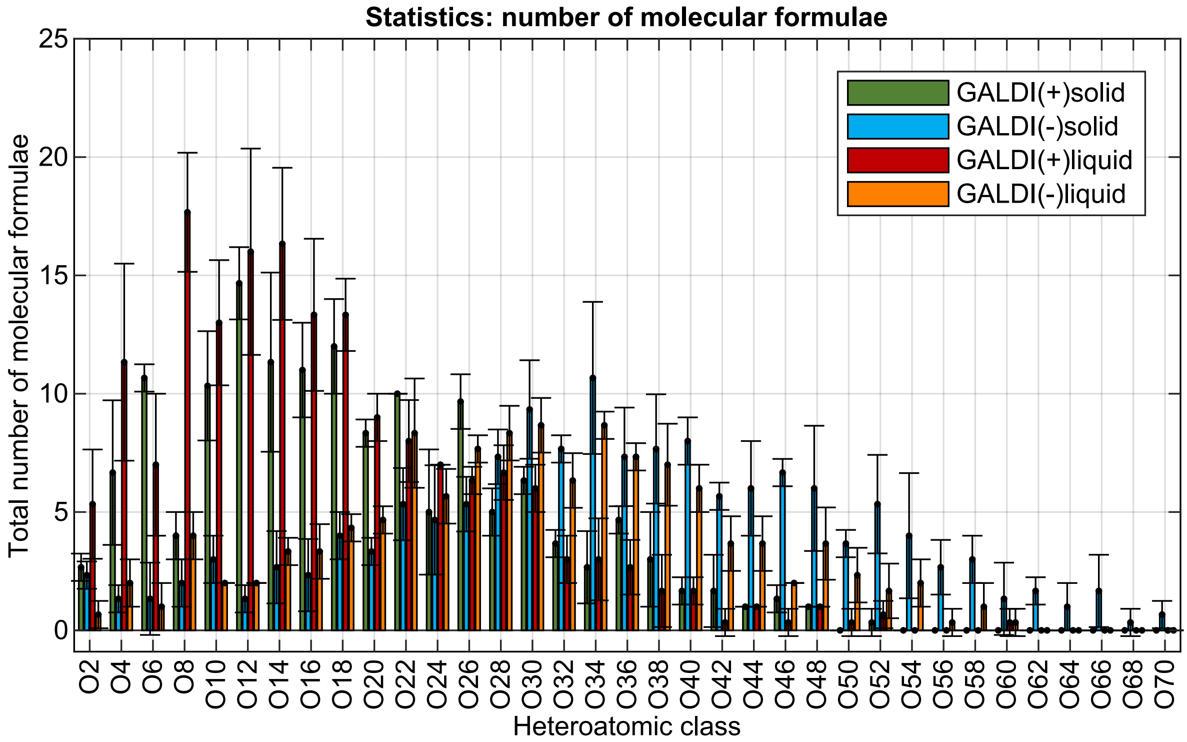

Figure S14: Statistical overview of the molecular formula evaluation for the four developed analysis methods, illustrating the total number of molecular formulae for selected oxygen containing classes between O_2 and O_{70}.

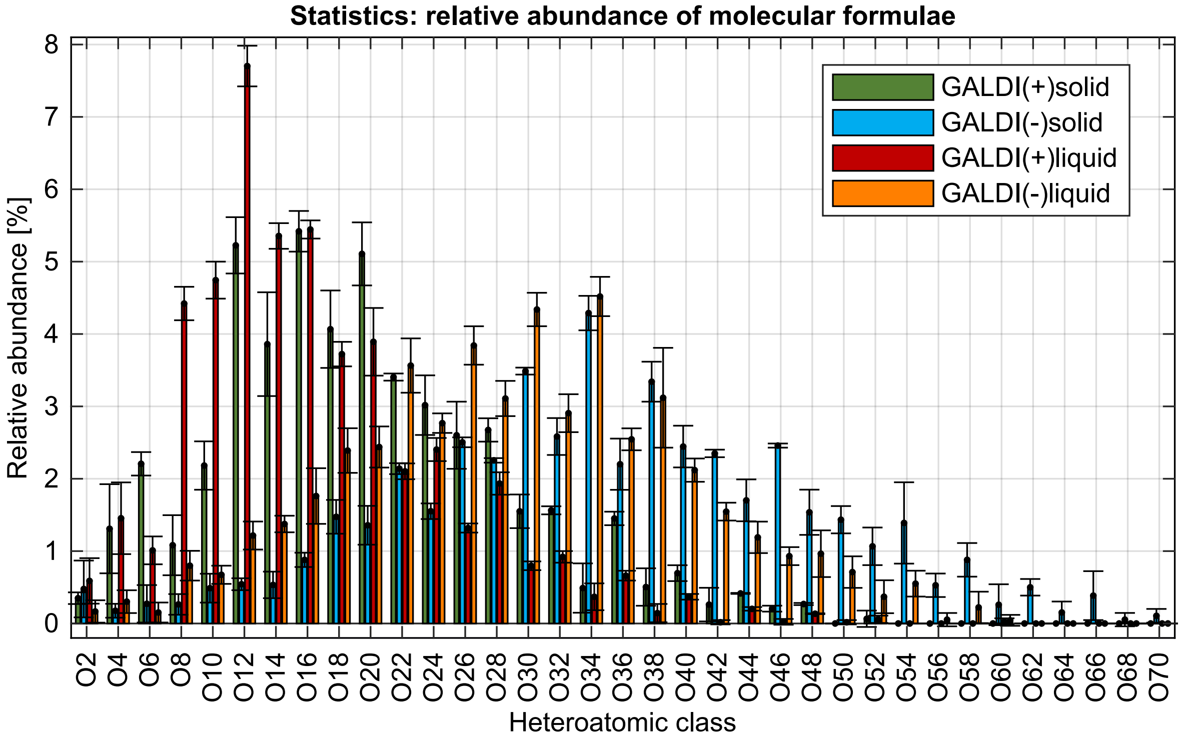

Figure S15: Statistical overview of the molecular formula evaluation for the four developed analysis methods, illustrating the relative abundance of molecular formulae for selected oxygen containing classes between O_2 and O_{70}.

S4 Van Krevelen plots of the liquid-state GALDI(+/−)-FT-ICR-MS methods and the van Krevelen based relative overall composition for all methods

Figure S16 shows the van Krevelen plots of the xylan sample for the liquid-state analysis routines. Herein the key results align with those presented in the main part of the paper, which focuses on the solid-state methods.

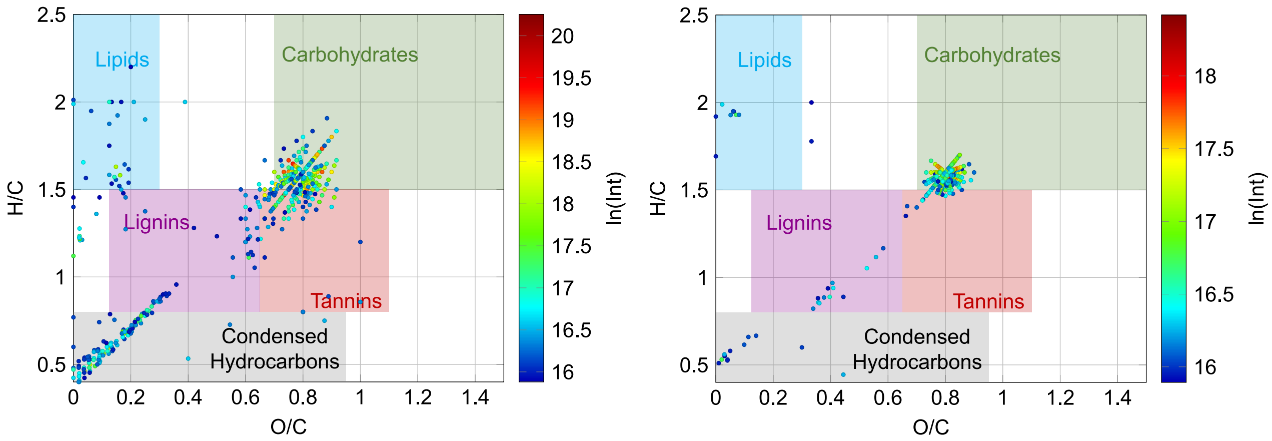

(a) GALDI(+)liquid-FT-ICR-MS

(b) GALDI(−)liquid-FT-ICR-MS

Figure S16: van Krevelen plots of the xylan sample analyzed using liquid-state GALDI-FT-ICR-MS. The observed intensities are presented logarithmic and color-coded (blue: low intensity, green to yellow: medium intensity, red: high intensity).The boundaries for the compound class regions are defined according to Ayala-Ortiz et al.[1]

Based on the van Krevelen data the percentage (intensity-weighted) of the assigned molecular formulae in the different compound classes could be determined. As illustrated in Figure S17 and demonstrated in Table S2, the majority of the assigned molecular formulae, as expected, are classified as carbohydrates (positive: approx. 60 %, negative: approx. 90 %). It is also probable that the data points in the tannin region, due to their position, could be assigned to the xylan oligomers in the sample. Due to dehydration reactions during the ionization, the H/C and O/C ratios of the oligomers decrease slightly. In addition, it should be noted that the established boundaries for these regions represent mean values and are not to be considered absolute.

Table S2: Percentage of assigned molecular formulae in the different compound classes based on the van Krevelen data for all four developed methods.

compound class	GALDI(+)solid	GALDI(−)solid	GALDI(+)liquid	GALDI(-)liquid
Others	0.85 %	0.00 %	0.00 %	0.00 %
Condensed hydrocarbons	25.85 %	1.06 %	18.86 %	1.57 %
Tannins	4.99 %	2.29 %	10.31 %	2.77 %
Lignins	4.89 %	6.99 %	3.91 %	1.88 %
Unsaturated hydrocarbons	0.45 %	0.12 %	0.97 %	0.00 %
Carbohydrates	60.67 %	86.92 %	62.27 %	92.26 %
Amino sugars	0.41 %	0.00 %	1.63 %	0.00 %
Proteins	0.24 %	0.08 %	0.07 %	0.22 %
Lipids	1.65 %	2.55 %	1.98 %	1.29 %

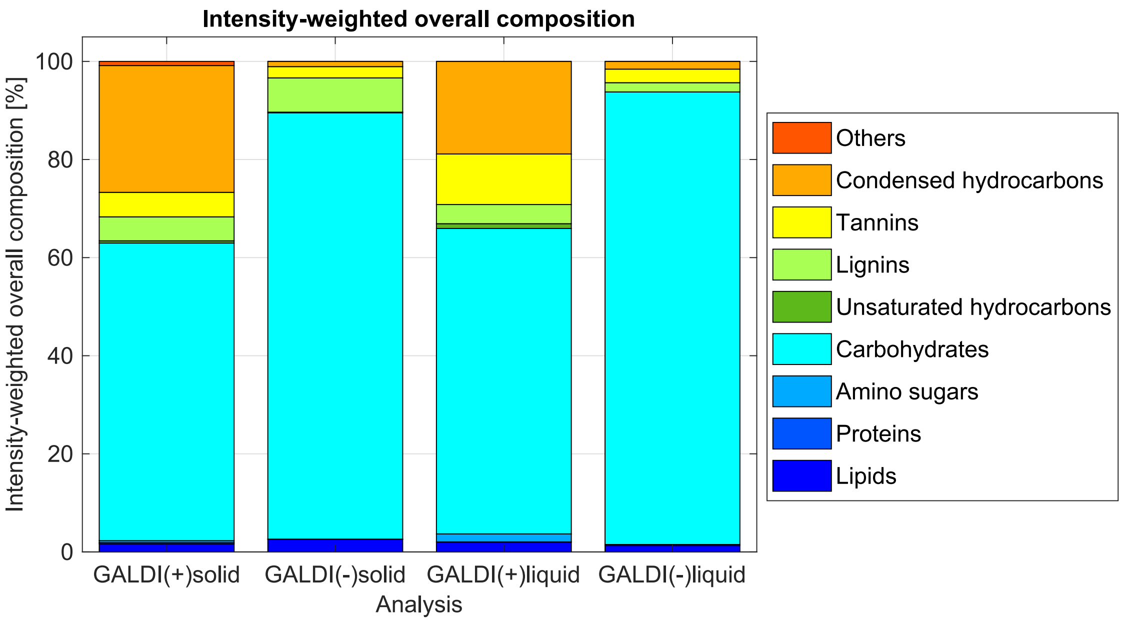

Figure S17: Graphical representation of the percentage of assigned molecular formulae in the different compound classes based on the van Krevelen data for all four developed methods.

S5 n_C-DBE plots for the solid-state analysis routines

Figure S18 illustrates the n_C-DBE plots of the beechwood xylan for the solid-state analysis routines in the positive and negative ionization mode, with the number of oxygen (n_O) plotted on the z axis (color map). The DBE thereby represents the number of double bonds and rings in a molecule and is calculated directly from the molecular formula via equation (1) [2].

$$\mathrm{DBE} = \frac{2 \cdot c - h + n + 2}{2} \tag{1}$$

Herein c is the number of carbon atoms, n is the number of nitrogen atoms and h is the number of hydrogen atoms, present in the molecule. The n_C value, in turn, serves as measure for the chain length or molecule size.

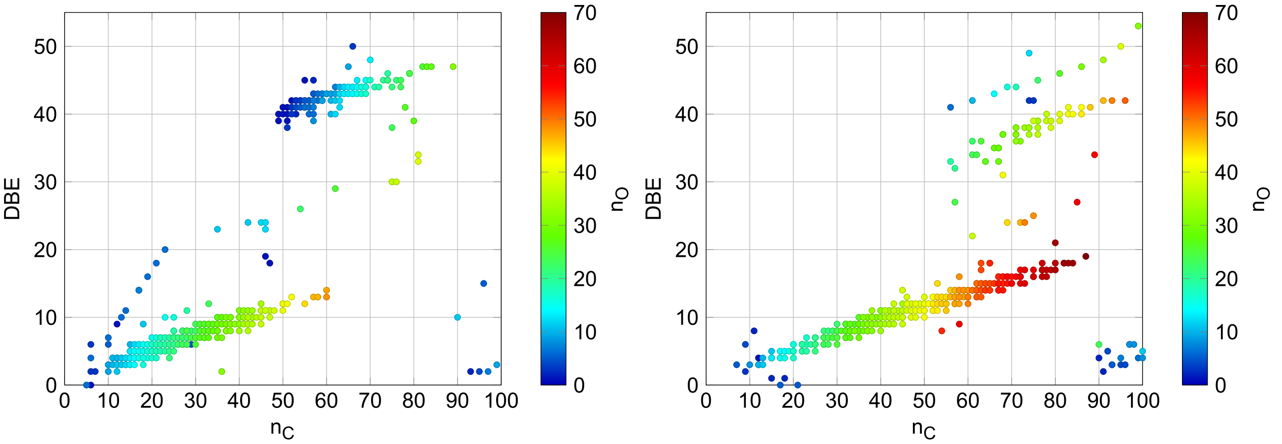

(a) GALDI(+)solid-FT-ICR-MS (b) GALDI(−)solid-FT-ICR-MS

Figure S18: n_C-DBE-n_O plots for the solid-state GALDI-FT-ICR-MS for the positive and negative ionization mode. The number of oxygen atoms in each molecule is visualized by a color bar ranging from 0 to 70 for both ionization modes.

In the n_C-DBE plots two main groups of data points are noticeable. The first one is characterized by relatively low values for the DBE, while the number of carbon atoms covers a relatively wide range. This region is characteristic for the xylooligomers in the xylan sample. The two ionization modes thereby differ in terms of the size of the oligomers that can be ionized. This results in a smaller group of data points for the positive ionization mode (n_C = 6 to 60; DBE = 2 to 14) compared to the negative ionization mode (n_C = 7 to 87; DBE = 2 to 19). The number of oxygen atoms also differs between the ionization modes corresponding to the oligomer sizes. For the positive ionization mode a smaller oxygen content is evident (n_O = 8 to 48) compared to the negative ionization mode (n_O = 8 to 70).

As demonstrated in Figure S18, the second group of data points is located at higher n_C and DBE values. The DBE values thereby are modestly elevated in the positive ionization mode (38 to 48) relative to the negative ionization mode (30 to 42). Conversely, this trend is reversed for the n_C values. In this case, the values for the negative ionization mode are slightly higher (60 to 90) than for the positive ionization mode (50 to 80). The predominant difference between the ionization modes, however, is the quantity of oxygen atoms (n_O) present in these molecules. While the oxygen content is low to moderate (1 to 25) for the positive ionization mode, it was determined to be moderate to high (20 to 50) in the negative ionization mode. Thus the results indicate that the second group of molecules in the positive ionization mode displays increased unsaturation, reduced size, and decreased oxygen functionalization compared to those in the negative ionization mode. Consequently, these data points do not originate from the same substances, as already described in the main part of the paper. The presence of these compounds could be attributed to impurities present in the sample, which might have been generated during the production process or were already contained in the sample and not removed during the purification stage.

S6 Oligomer series labeled mass spectra of the solid-state GALDI-FT-ICR-MS methods in both ionization modes

Figures S19(a) and S19(b) provide a larger version of the mass spectra of a selection of homologous oligomer series for the solid-state methods in both ionization modes that are presented in the main part of the paper.

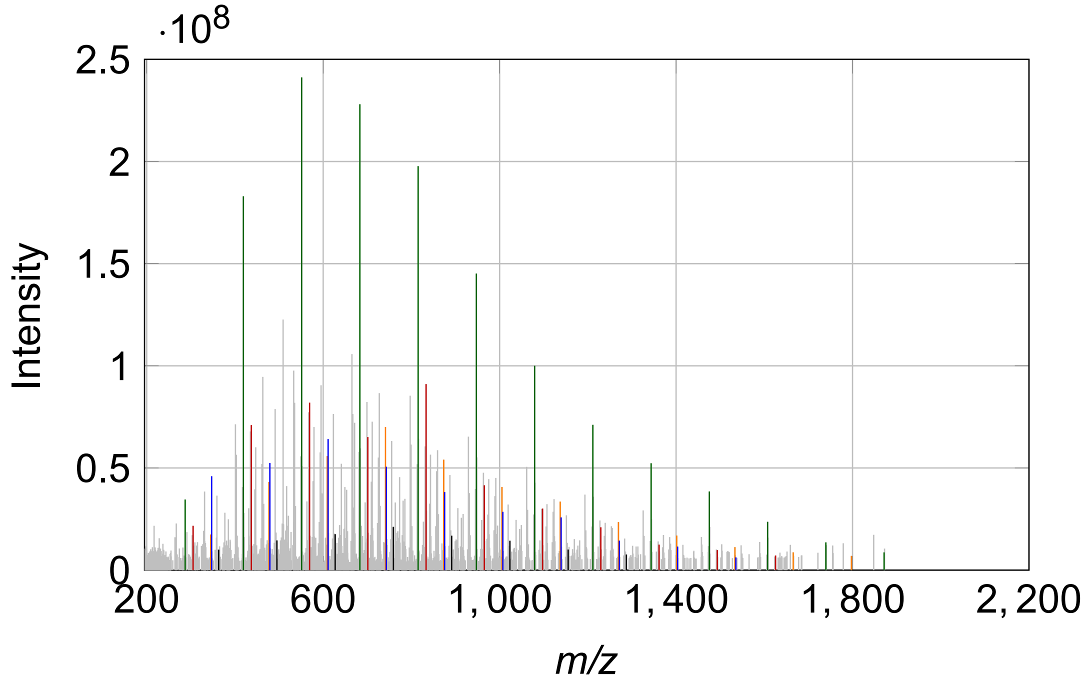

(a) GALDI(+)solid-FT-ICR-MS.

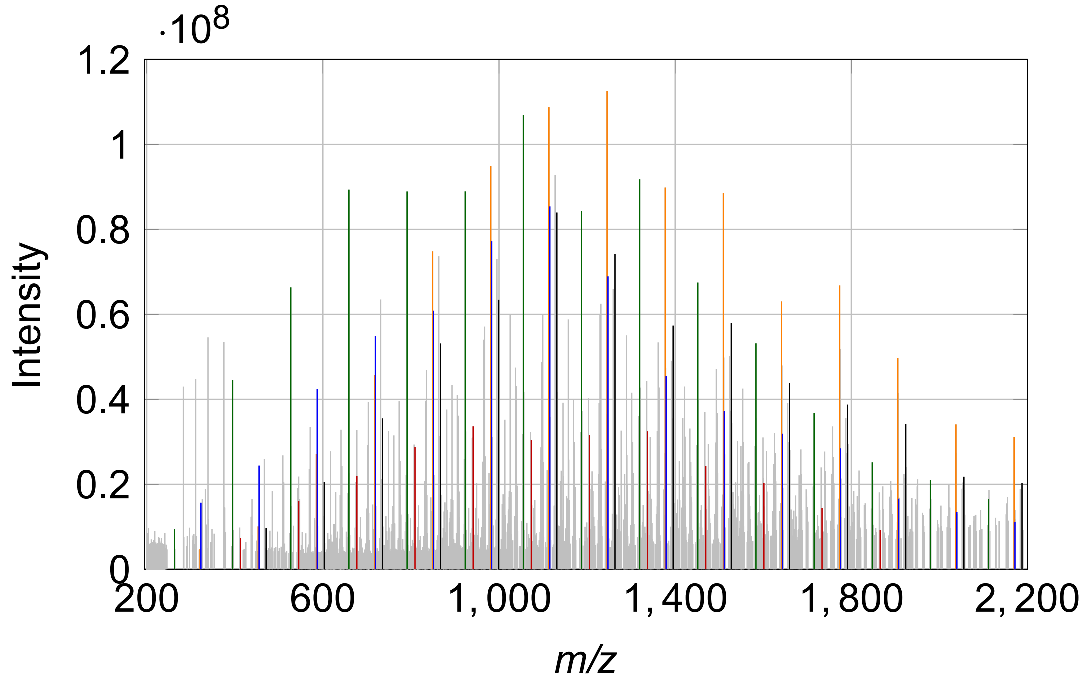

(b) GALDI(−)solid-FT-ICR-MS.

Figure S19: Mass spectra for both ionization modes for the solid-state analysis routines are presented. The colors of the signals correspond to the following oligomer series: red = [X_n], green = [$X_n - H_2O$], blue = [X_nAc], orange = [X_n(MeGlcA) − H_2O], and black = [X_n(MeGlcA)]; where X corresponds to xylose, Ac to acetylated species and MeGlcA to 4-O-methylglucuronic acid.

S7 n_C-DBE and RKM-m/z plots of the liquid-state GALDI(+/−)-FT-ICR-MS methods

Figure S20 shows the n_C-DBE plots of the beechwood xylan for the liquid-state analysis routines. The key results align with those presented in section S5 for the solid-state methods.

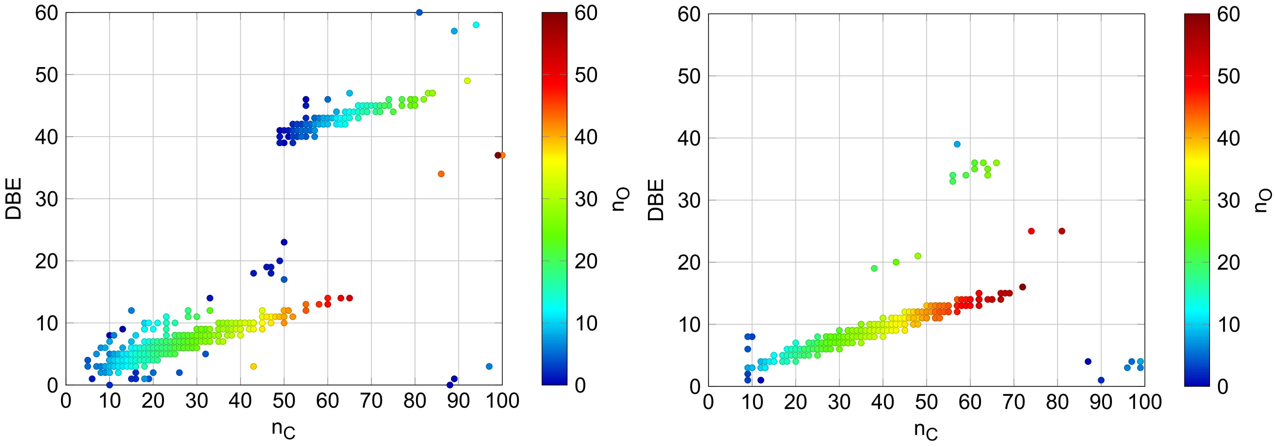

(a) GALDI(+)liquid-FT-ICR-MS

(b) GALDI(−)liquid-FT-ICR-MS

Figure S20: n_C-DBE-n_O plots for the liquid-state GALDI-FT-ICR-MS for the positive and negative ionization mode. The number of oxygen atoms in each molecule is visualized by a color bar ranging from 0 to 60 for both ionization modes.

Additionally, Figures S22(a) and S22(b) present the mass spectra of a selection of homologous oligomer series for the liquid-state methods in both ionization modes, while Figures S21(a) and S21(b) illustrate the corresponding RKM-m/z plots. These figures comply with the data presented in the main part of the paper. The m/z ranges and the corresponding structural proposals for both ionization modes are presented in Table S3.

Table S3: Summary of the selected oligomer series for the negative and positive ionization mode for the liquid-state analysis routines. In this X corresponds to xylose, Ac to acetylated species and MeGlcA to 4-O-methylglucuronic acid.

ionization mode	m/z range	structural proposal
negative	281.087859 to 1,469.467986	$[X_n]$
	263.077238 to 1,847.583609	$[X_n - H_2O]$
	323.098357 to 1,907.605475	$[X_nAc]$
	321.082722 to 2,037.632543	$[X_n(MeGlcA) - H_2O]$
	339.093264 to 1,923.600571	$[X_n(MeGlcA)]$
positive	305.084305 to 1,625.507108	$[X_n]$
	287.073751 to 1,739.538588	$[X_n - H_2O]$
	347.094868 to 1,535.475702	$[X_nAc]$
	345.079221 to 1,533.459918	$[X_n(MeGlcA) - H_2O]$
	363.089775 to 1,155.343792	$[X_n(MeGlcA)]$

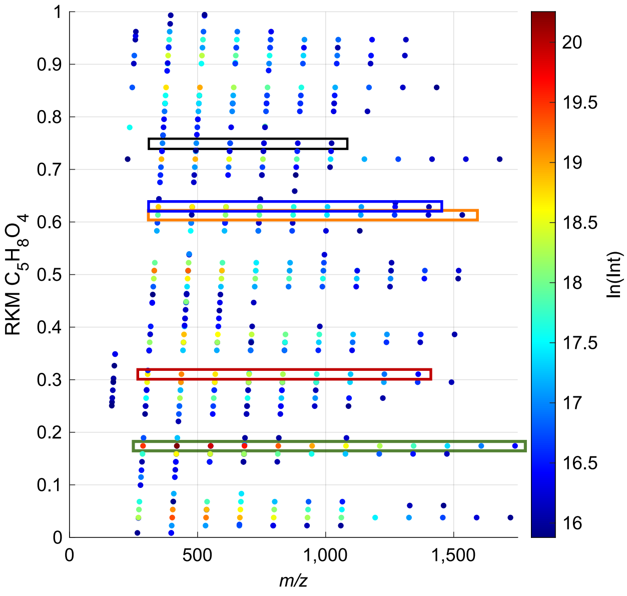

(a) RKM-m/z -plot for GALDI(+)liquid-FT-ICR-MS.

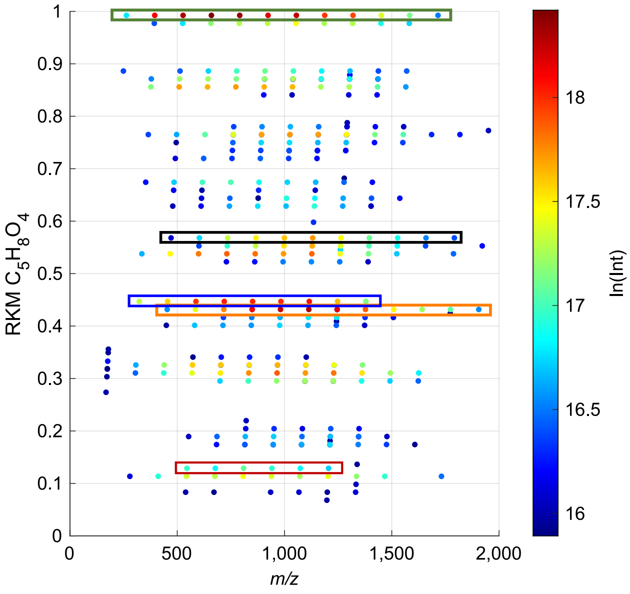

(b) RKM-m/z -plot for GALDI(−)liquid-FT-ICR-MS.

Figure S21: RKM-m/z plots for both ionization modes for the liquid-state analysis routines are presented. The observed intensities of the data points are presented logarithmic and color-coded (blue: low intensity, green to yellow: medium intensity, red: high intensity) The markings correspond to the following oligomer series: red = $[X_n]$, green = $[X_n - H_2O]$, blue = $[X_nAc]$, orange = $[X_n(MeGlcA) - H_2O]$, and black = $[X_n(MeGlcA)]$; where X corresponds to xylose, Ac to acetylated species and MeGlcA to 4-O-methylglucuronic acid.

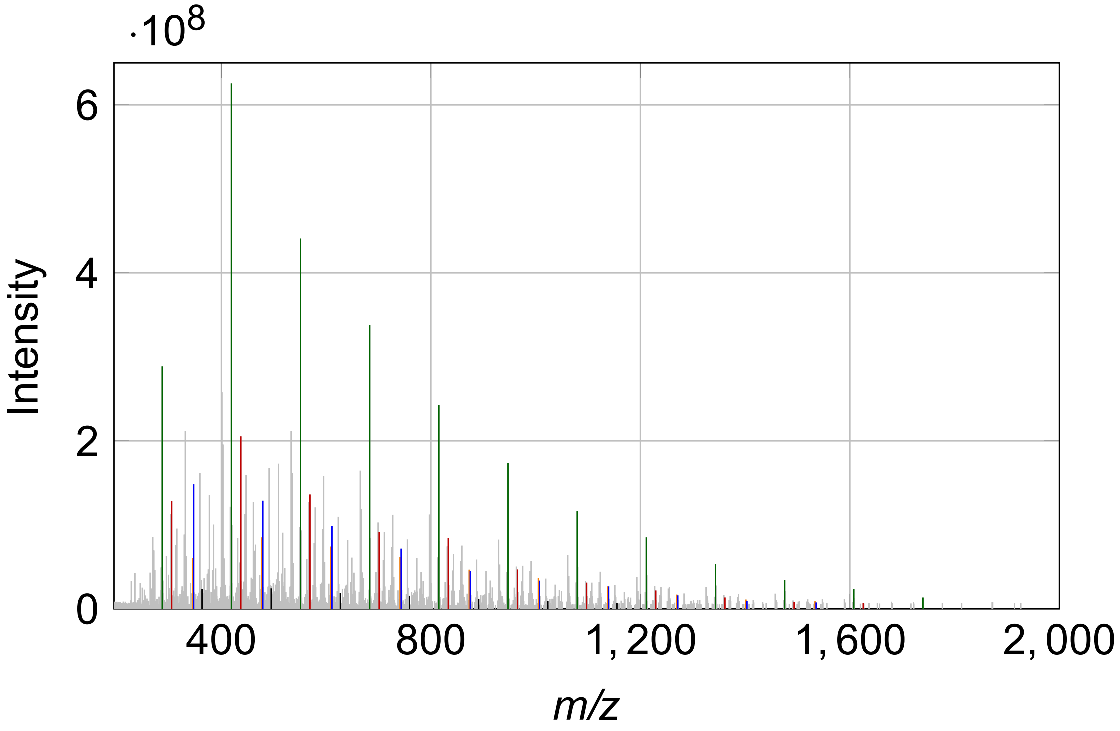

(a) GALDI(+)liquid-FT-ICR-MS.

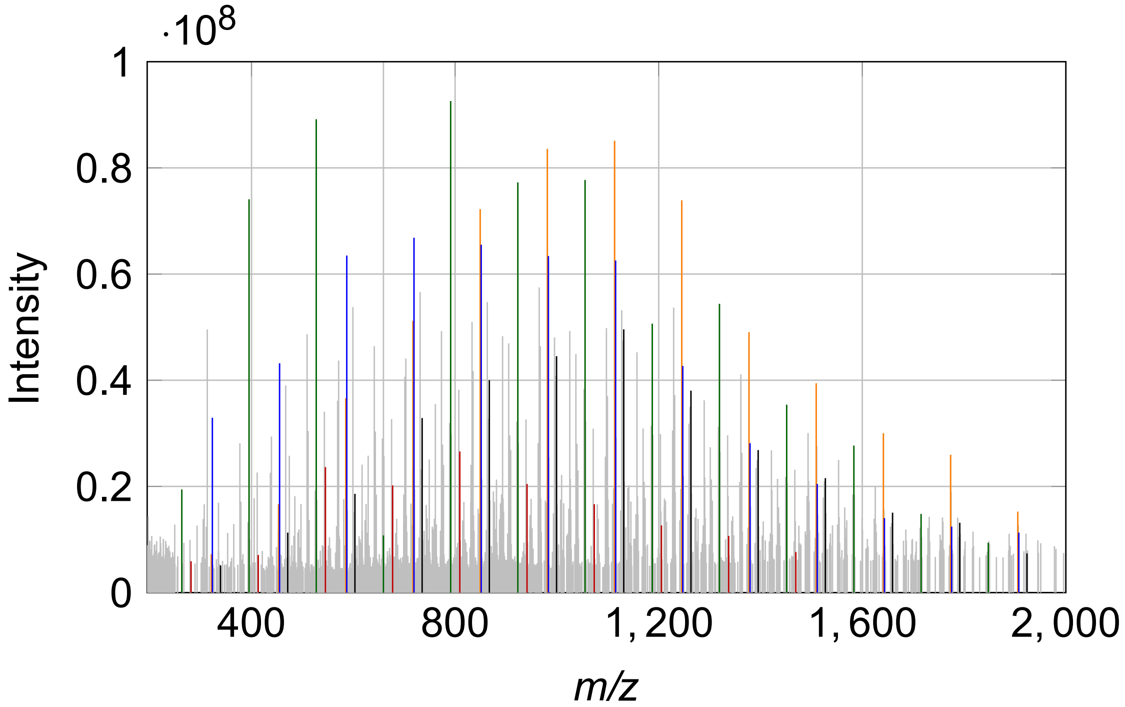

(b) GALDI(−)liquid-FT-ICR-MS.

Figure S22: Mass spectra for both ionization modes for the liquid-state analysis routines are presented. The colors of the signals correspond to the following oligomer series: red = $[X_n]$, green = $[X_n - H_2O]$, blue = $[X_nAc]$, orange = $[X_n(MeGlcA) - H_2O]$, and black = $[X_n(MeGlcA)]$; where X corresponds to xylose, Ac to acetylated species and MeGlcA to 4-O-methylglucuronic acid.

References

[1] Ayala-Ortiz, C.; Graf-Grachet, N.; Freire-Zapata, V.; Fudyma, J.; Hildebrand, G.; AminiTabrizi, R.; Howard-Varona, C.; Corilo, Y. E.; Hess, N.; Duhaime, M. B.; Sullivan, M. B.; Tfaily, M. M. MetaboDirect: an analytical pipeline for the processing of FT-ICR MS-based metabolomic data. *Microbiome* **2023**, *11*.

[2] Liu, F.; Fan, M.; Wei, X.; Zong, Z. Application of mass spectrometry in the characterization of chemicals in coal-derived liquids. *Mass Spectrom. Rev.* **2016**, *36*, 543–579.
